# Loss of Fgr41 in *Candida albicans* attenuates virulence and increases proinflammatory immune responses in a manner that is dependent on β(1,3)-glucan but not dectin-1

**DOI:** 10.1128/iai.00523-25

**Published:** 2026-03-27

**Authors:** Ainsley E. King, Mikayla M. Mangrum, Sarah J. Kauffman, Kaley E. Taylor, Duncan Arnold, Andrew S. Wagner, Todd B. Reynolds

**Affiliations:** 1 Department of Microbiology, University of Tennessee, Knoxville, Tennessee, USA; University of Pennsylvania Perelman School of Medicine, Philadelphia, Pennsylvania, USA

**Keywords:** macrophage, cell wall, ß-glucan, dectin-1, Fgr41, *Candida*

## Abstract

β(1, 3)-glucan is an essential cell wall polysaccharide in the fungal pathogen *Candida albicans* and is also an important immunogenic epitope for host recognition. This polymer is normally masked from immune recognition by a layer of mannosylated glycoproteins on the outer cell wall, but it can become exposed in certain conditions. Prior work showed that the putative secretory protein Fgr41 is downregulated in conditions that increase β(1, 3)-glucan exposure, including caspofungin treatment or activation of the Cek1 MAP kinase pathway. Disruption of *FGR41* increases *C. albicans* β(1, 3)-glucan unmasking and reduces kidney fungal burden in murine systemic infections in an immune-dependent manner. We tested the impact of *FGR41* on immune evasion by measuring tumor necrosis factor alpha (TNF-α) secretion from murine macrophages *in vitro*. The *fgr41Δ/Δ* mutant elicited ~4 times more TNF-α from macrophages than wild-type. Antibody neutralization of dectin-1 did not significantly reduce TNF-α released from macrophages challenged with the *fgr41Δ/Δ* strain. However, an inhibitory, soluble β-glucan (laminarin) significantly reduced *fgr41Δ/Δ-*induced stimulation, suggesting that macrophage recognition of the *fgr41Δ/Δ* mutant is driven by the detection of exposed β(1, 3)-glucan by another receptor. When assessed *in vivo*, the attenuated virulence observed for the *fgr41Δ/Δ* strain in wild-type mice was similar in dectin-1^-/-^ mice, indicating that a non-dectin-1 mechanism recognizes the *fgr41Δ/Δ* mutant *in vivo*. Genetic data suggest that the role of *FGR41* in immune evasion is partially dependent on the cell wall β-glucanase *ENG1*. Overexpression of *ENG1* partially suppresses *fgr41Δ/Δ-*induced unmasking and TNF-α stimulation, but the opposite is not true, indicating that these proteins may work together.

## INTRODUCTION

The opportunistic fungal pathogen *Candida albicans* is a common cause of mucosal and systemic infections, particularly in immunocompromised patients (1–3). A healthy innate immune system is generally able to prevent oral or systemic infections, but treatment with immunosuppressants such as chemotherapy drugs allows the fungus an opportunity to proliferate and cause disease (4). Systemic candidiasis is of particular interest, as it leads to dissemination of the fungus throughout the body and is associated with mortality rates exceeding 30% (5, 6). While several different classes of antifungals are available for the treatment of systemic infections, these mortality rates persist due to a number of factors, including antifungal resistance and toxicity to the host (7, 8). Therapies combining traditional antifungals with immunotherapy have been increasingly considered an alternative treatment method, but recombinant cytokine therapy is the only form of immunotherapy currently approved for clinical use (4, 9). The development of additional immunotherapeutic treatments is hindered by an incomplete understanding of the complex interactions between *C. albicans* and the host immune system (4). Investigation into the molecular mechanisms involved in *C. albicans* immunity may promote the development of immunotherapeutic treatment strategies.

Upon infection, neutrophils and macrophages can recognize *C. albicans* using a variety of pattern recognition receptors (PRRs) present on the surfaces of innate immune cells. These PRRs detect specific pathogen-associated molecular patterns (PAMPs) on the surface of *C. albicans* (10). As the outermost cellular layer, the *C. albicans* cell wall contains many PAMPs recognized by innate immune cells. The cell wall is composed primarily of an interconnected network of polysaccharides, including a basal layer of chitin, β(1, 3)-glucan, and β(1, 6)-glucan (4). On top of these structural polymers sits a layer of glycosylated proteins carrying *N*-linked and *O*-linked glycans, as well as phospholipomannans (11). The outer mannoprotein layer is recognizable by some PRRs, including some toll-like receptors (TLRs) such as TLRs 2, 4, and 6 (12–15). Additionally, C-type lectin receptors (CLRs) such as dectin-2, DC-SIGN/SIGNR1, and mannose receptor (MR) can also recognize mannans (15, 16). However, the mannoproteins on the surface mask the lower layers of the cell wall from immune recognition, particularly β(1, 3)-glucan, which is highly immunogenic when exposed (4, 17–20). Several different environmental conditions are known to alter β(1, 3)-glucan exposure, including exposure to lactate, acidic pH, or the echinocandin class of antifungals (20–25). Recognition of exposed β-glucan stimulates a robust immune response and leads to increased fungal clearance (17, 18, 26). The CLR dectin-1 is thought to be primarily responsible for the recognition of exposed β(1, 3)-glucan in the *C. albicans* cell wall, although other receptors, such as complement receptor 3 (CR3), have been described (27–30). Dectin-1 on the surface of bone marrow-derived macrophages (BMDMs) preferentially binds to areas of unmasking, suggesting a direct interaction (24).

In addition to environmental cues, genetic changes can lead to *C. albicans* β(1, 3)-glucan exposure. A number of genes have been shown to play a role in unmasking, and mutations that induce high levels of β(1, 3)-glucan exposure are frequently correlated with dectin-1 recognition. For example, loss of the α−1,6-mannosyltransferase Mnn10 caused both an increase in β(1, 3)-glucan exposure and a dectin-1-dependent increase in macrophage stimulation (31). A knockout of the phosphatidylserine synthase Cho1 also exhibited greater unmasking and dectin-1 binding than wild type (32). The endo-β(1, 3)-glucanase Eng1, which is required for cell separation in both *C. albicans* and other yeasts, is thought to regulate β(1, 3)-glucan exposure by hydrolyzing β(1, 3)-glucan at the bud neck during division (33–36). An *eng1Δ/Δ* mutant also showed both an increase in dectin-1 binding and a dectin-1-dependent stimulation of the proinflammatory cytokine tumor necrosis factor alpha (TNF-α) from BMDMs (37).

The putative secreted protein Fgr41 is another effector shown to play a role in β(1, 3)-glucan exposure (38, 39). It is downregulated in multiple unmasking conditions, including hyperactivation of the MAP3 kinase *STE11* (*STE11^ΔN467^*) and caspofungin treatment (40, 41). Deletion of *FGR41* resulted in an increase in β(1, 3)-glucan exposure, and its overexpression in the highly unmasked *STE11^ΔN467^* background reduced β(1, 3)-glucan exposure to a level closer to that of wild type (39). Interestingly, the *FGR41* mutant shares several of these phenotypes with an *ENG1* mutant, including increased β(1, 3)-glucan exposure localized to bud necks and scars and downregulation in unmasking conditions (37, 40, 42). Both *fgr41Δ/Δ* and *eng1Δ/Δ* also exhibit a defect in cell separation (34, 37, 39). Although this could suggest a relationship between Eng1 and Fgr41, no investigation has been done in this area. Furthermore, Fgr41 remains largely uncharacterized; bioinformatic predictions suggest that it may be cell wall-associated and play some role in adhesion, but no further functional characterization has been performed (38, 43). This lack of experimental evidence makes it difficult to postulate what role Fgr41 may be playing in the cell, or what type of interactions it could have with Eng1.

Although its cellular role is not fully characterized, Fgr41 is known to be important for *C. albicans* virulence. The *fgr41Δ/Δ* mutant was found to have attenuated colonization in a zebrafish burn wound infection model (44). In a murine model of systemic infection, *fgr41Δ/Δ* showed reduced kidney fungal burden compared to wild type at 4 days post-infection (dpi). This difference was eliminated when the mice were immunosuppressed, suggesting that the immune system plays a key role in the virulence defect of the *fgr41Δ/Δ* mutant (39). However, a direct link between the increased β(1, 3)-glucan exposure of *fgr41Δ/Δ* and its virulence attenuation has never been elucidated, nor has dectin-1 been directly implicated in this response. Here, we show that the *fgr41Δ/Δ* mutant stimulates high levels of TNF-α from RAW 264.7 murine macrophages in a manner that requires recognition of exposed β(1, 3)-glucan but is independent of dectin-1. Furthermore, we show that the *fgr41Δ/Δ* mutant exhibits severely attenuated virulence in a systemic mouse model regardless of the presence of dectin-1, indicating that another immune receptor is responsible for recognizing exposed β(1, 3)-glucan on the cell surface.

## RESULTS

### Overexpression of *ENG1* partially rescues unmasking and macrophage TNF-α release in the *fgr41Δ/Δ* mutant

In order to investigate the relationship between Fgr41 and Eng1, a series of mutants was created that alter the expression of each gene (Table S3). An *FGR41* knockout mutant, an *FGR41* overexpression strain, and an *FGR41* complement strain were previously created by our lab (39). An *ENG1* knockout mutant (*eng1Δ/Δ*) and an *ENG1/FGR41* double mutant (*eng1Δ/Δ fgr41Δ/Δ*) were generated using CRISPR-Cas9 (45). We then created an *ENG1* overexpression strain and a complement strain of the *eng1Δ/Δ* mutant. Finally, combination mutants were made, in which *ENG1* was knocked out and *FGR41* was overexpressed (*eng1Δ/Δ FGR41^oe^*), or *FGR41* was knocked out and *ENG1* was overexpressed (*fgr41Δ/Δ ENG1^oe^*).

**Fig 1:**
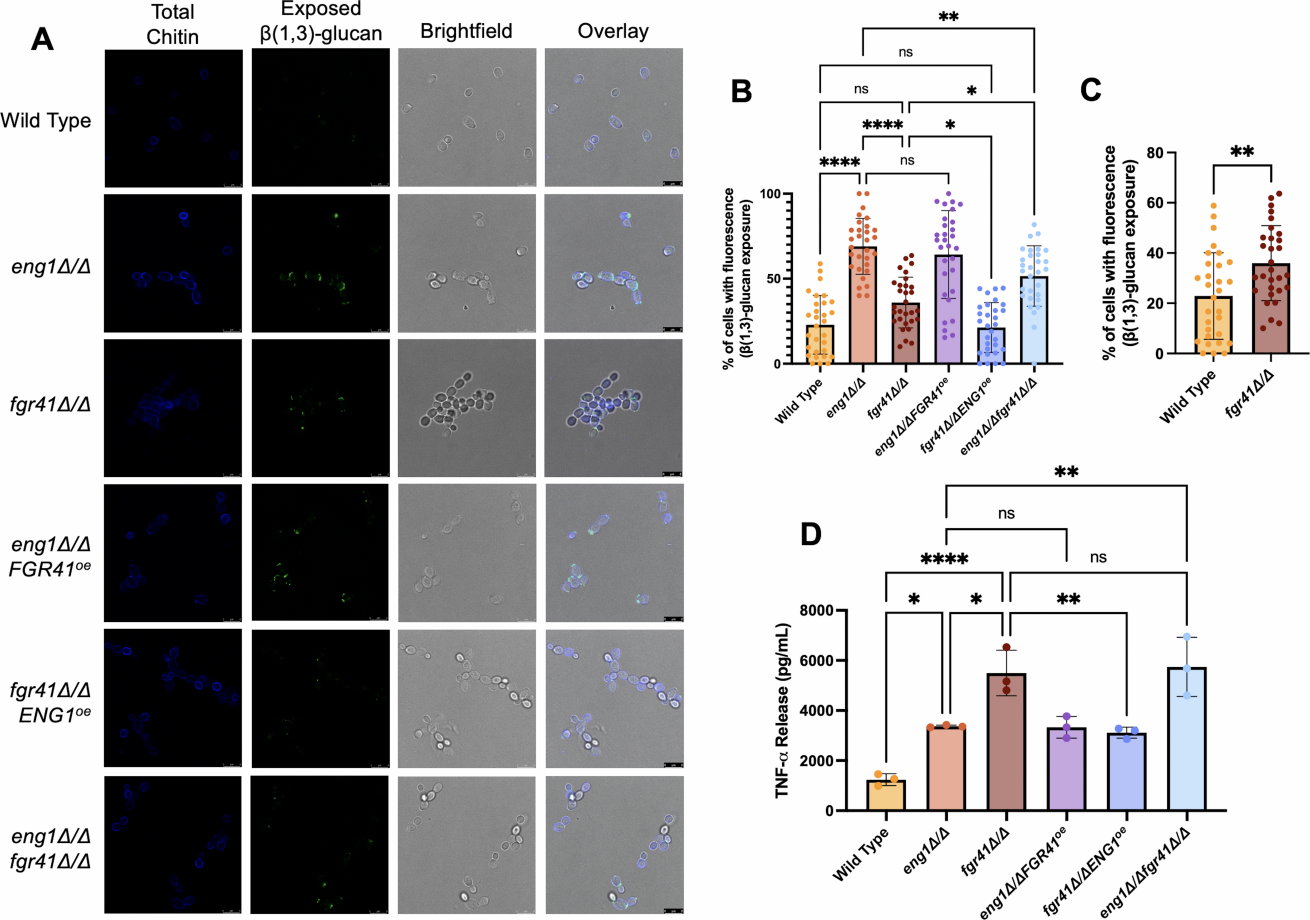
*FGR41* knockout-induced β(1, 3)-glucan exposure and macrophage TNF-α release are decreased by *ENG1* overexpression. (**A**) Cells were stained for confocal microscopy with a primary antibody to β(1, 3)-glucan and a secondary antibody conjugated to Alexa Fluor 488 to visualize unmasking, and CFW to visualize total chitin. A total of 30 images was taken of each strain across three biological replicates. Scale bar = 8 µm. (**B**) Quantification of the exposed β(1, 3)-glucan in panel A. A global threshold of 60 was applied to each exposed β(1, 3)-glucan image, and the percentage of cells showing fluorescence in each image was determined using Fiji ImageJ. ****P < 0.0001, **P < 0.01, *P < 0.05, by one-way analysis of variance (ANOVA). (**C**) The wild type and *fgr41Δ/Δ* bars from panel B were analyzed in isolation, which revealed a significant difference (**P < 0.01 by one-way ANOVA). (**D**) RAW 264.7 macrophages were co-incubated with UV-inactivated *C. albicans* for 4 h, and the resulting supernatants were analyzed for TNF-α concentrations via ELISA. The experiment was performed three times with three technical replicates each time; each point represents the average of three technical replicates. ****P < 0.0001, **P < 0.01, *P < 0.05, by one-way ANOVA, ns = not significant.

To examine the levels of β(1, 3)-glucan exposure and total chitin in these strains, we stained each mutant with an antibody to β(1, 3)-glucan and for chitin with calcofluor white (CFW) and imaged the cells using confocal microscopy. As expected, both the *eng1Δ/Δ* and *fgr41Δ/Δ* mutants showed higher levels of β(1, 3)-glucan exposure than wild type (Fig. 1A through C), with the *eng1∆/∆* mutant exhibiting a higher percentage of cells with exposed β(1, 3)-glucan than the *fgr41∆/∆* mutant. This exposure was concentrated at the bud necks and scars, as seen previously (37, 39). The *eng1Δ/Δ FGR41^oe^* strain showed no difference in unmasking when compared with the *eng1Δ/Δ* strain (Fig. 1B). In contrast, when *ENG1* was overexpressed in the *fgr41Δ/Δ* strain, there were significantly fewer cells with exposed β(1, 3)-glucan than in the *fgr41Δ/Δ* strain alone. This suggests that the overproduction of Eng1 is able to partially compensate for the loss of Fgr41 and restore unmasking closer to wild type levels, but not vice versa. Furthermore, the *eng1Δ/Δ fgr41Δ/Δ* double mutant showed less unmasking than the *eng1Δ/Δ* mutant but significantly more than the *fgr41Δ/Δ* mutant. Altogether, this could indicate that Eng1 is epistatic to Fgr41. The other major phenotype was a cell separation defect, and the *fgr41Δ/Δ* mutant shows a larger defect than the *eng1Δ/Δ* mutant (Fig. 1A). This defect is not corrected by the overexpression of *ENG1* or worsened by the deletion of *ENG1*, indicating that this phenotype is not directly related to their shared impact on β(1, 3)-glucan exposure.

Because β(1, 3)-glucan exposure and immune stimulation are frequently correlated, we next wanted to determine the ability of our mutants to stimulate a proinflammatory immune response (46). To do this, each mutant was UV-inactivated to prevent hyphal growth and co-incubated with RAW 264.7 murine macrophages for 4 h. Levels of the proinflammatory cytokine TNF-α were measured from the supernatant by ELISA, and it was found that both the *eng1Δ/Δ* and *fgr41Δ/Δ* strains induced a significantly higher level of TNF-α release than wild type. The *eng1Δ/Δ* mutant induced around a 2.7-fold increase (Fig. 1D). Interestingly, macrophages challenged with the *fgr41Δ/Δ* mutant exhibited approximately a 4.4-fold increase in TNF-α release compared to wild type (Fig. 1D). This trend is opposite that of the unmasking data (Fig. 1), where the *eng1Δ/Δ* mutant was more unmasked than the *fgr41Δ/Δ* mutant. Complementation of each mutant reduced these levels significantly, while overexpression of each gene in the wild-type background had no impact on TNF-α stimulation (Fig. S1A).

We next measured if overexpression of either gene would suppress the immune response elicited by the opposite mutant. The TNF-α levels stimulated by the *eng1Δ/Δ FGR41^oe^* strain were similar to those of the *eng1Δ/Δ* strain (Fig. 1D). In contrast, the *fgr41Δ/Δ ENG1^oe^* strain significantly suppressed TNF-α release compared to the *fgr41Δ/Δ* parent. These trends do reflect those seen in the stain for exposed β(1, 3)-glucan (Fig. 1); the overexpression of *ENG1* can partially compensate for the loss of *FGR41*, and the reverse is not true. In contrast to the unmasking data, the TNF-α release stimulated by the double knockout was similar to that of *fgr41Δ/Δ* alone. Thus, at some level, *ENG1* appears to be epistatic to *FGR41* for TNF-α release as well.

### The cell separation defects of *eng1Δ/Δ* and *fgr41Δ/Δ* impact phagocytosis by macrophages

It has been previously reported that both the *eng1Δ/Δ* and *fgr41Δ/Δ* strains have a defect in cell separation (37, 39). It is also known that the large size of *C. albicans* hyphae inhibits macrophage phagocytosis and that hyphal killing can involve several macrophages attacking the same hypha (47). We therefore hypothesized that the large size of the yeast clumps formed in the *eng1Δ/Δ* strain and especially the *fgr41Δ/Δ* strain could play a role in inhibiting macrophage phagocytosis. Furthermore, we found discrepancies between the level of unmasking in our double mutant (Fig. 1A and B) and the level of TNF-α release it induced (Fig. 1D), suggesting that some other factor (such as clumping) may be contributing to immune stimulation.

**Fig 2:**
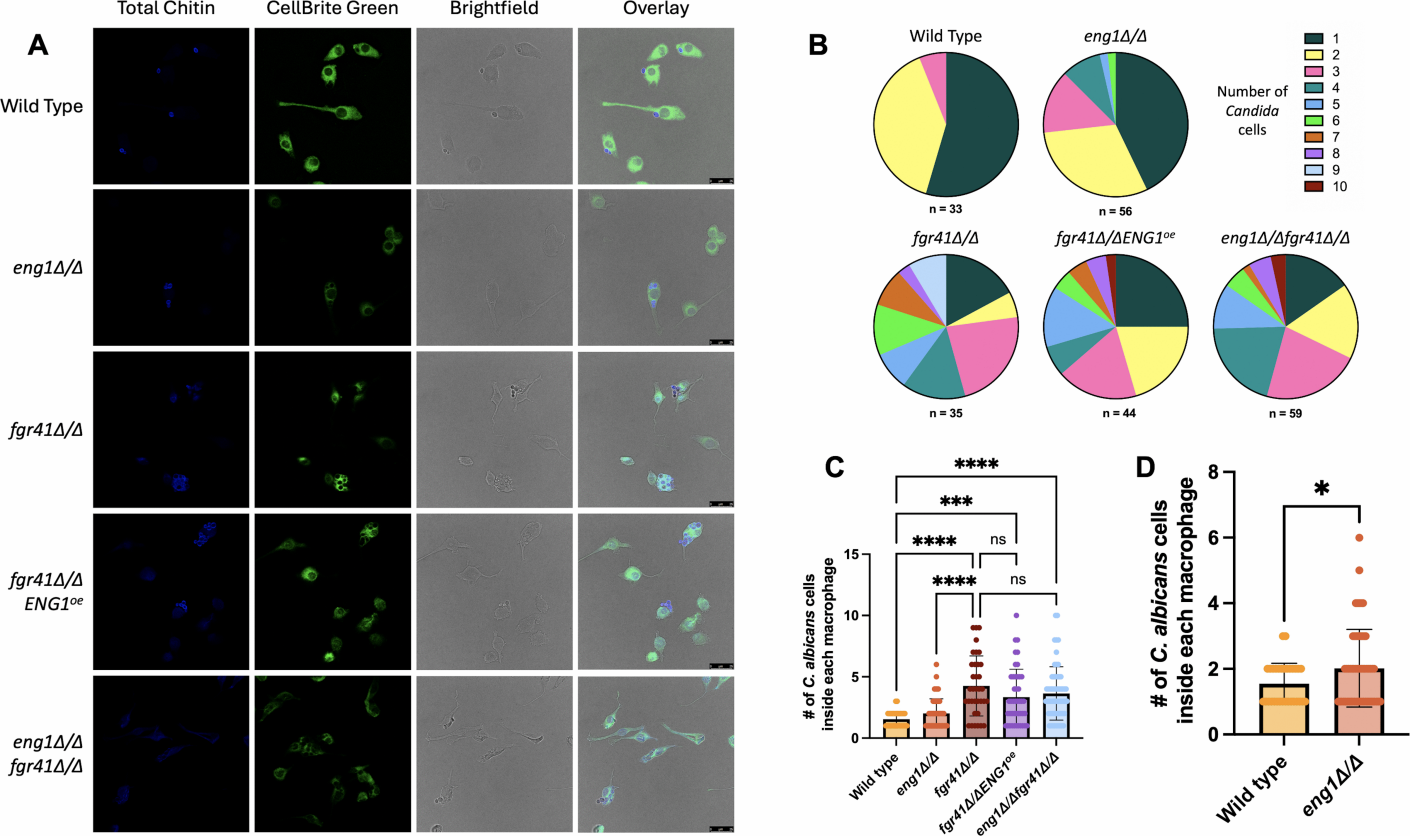
Cell separation defects lead to phagocytosis of larger clumps. RAW264.7 macrophages were co-incubated with UV-inactivated *C. albicans* for 2 h and fixed with 4% formaldehyde. Macrophages were stained with CellBrite Green, and *C. albicans* cells were stained with CFW. (**A**) Confocal microscopy images of macrophages co-incubated with wild type, *eng1Δ/Δ, fgr41Δ/Δ, fgr41Δ/ΔENG1^oe^,* or *eng1Δ/Δfgr41Δ/Δ* strains. Scale bar = 25 µm. (**B–D**) Quantification of panel A. A total of 40 images were analyzed for each strain. *C. albicans* cells were counted as being phagocytosed if they appeared internal to the macrophage and/or caused a gap in green fluorescence in the macrophage. (**B**) Percentages of macrophages containing each number of *C. albicans* cells in panel A. *n* = the total number of macrophages with at least one *C. albicans* cell inside them. Graphs including macrophages that had not phagocytosed any *C. albicans* are found in Fig. S2D. (**C**) The number of *C. albicans* cells inside each macrophage for each strain from panel A. Each point represents a macrophage that had phagocytosed at least one *C. albicans* cell. The number of macrophages represented in each bar corresponds to the *n* in panel B. ****P <0.0001, ***P < 0.001, by one-way ANOVA, ns = not significant. (**D**) The wild type and *eng1Δ/Δ* bars from panel C were analyzed in isolation, which revealed a significant difference (*P < 0.05, by Welch’s *t*-test).

To investigate this, we first looked at the clumping of each strain. Although the *fgr41Δ/Δ* mutant was clearly more clumped than wild type, we found no differences in the level of clumping between *fgr41Δ/Δ* and *fgr41Δ/Δ ENG1^oe^* strains (Fig. S2). Each of our single mutants, *fgr41Δ/Δ ENG1^oe^*, and the *eng1Δ/Δfgr41Δ/Δ* double mutant was then co-incubated with macrophages to observe the levels of phagocytosis. The *eng1Δ/Δ FGR41^oe^* strain was omitted because it did not show any differences from the *eng1Δ/Δ* mutant in unmasking or TNF-α release. The samples were fixed and stained for confocal microscopy (Fig. 2A). Clumps of cells were again evident in all of the mutants, with the clumps in the *fgr41Δ/Δ* strains being larger than those in the *eng1Δ/Δ* mutant. The number of *C. albicans* cells engulfed by each macrophage was counted, and the percentage of macrophages containing particular numbers of cells was calculated (Fig. 2B). The number of yeast cells inside each macrophage that had phagocytosed at least one *C. albicans* cell was plotted (Fig. 2C and D). Total macrophages counted, including those that had not engulfed any *C. albicans* cells, are shown in Fig. S2D. For wild-type *C. albicans*, most of the macrophages that contained yeast only contained one cell, and numbers higher than two were rare. In contrast, *eng1Δ/Δ* cells were found in significantly larger numbers inside macrophages (Fig. 2D), with up to six cells in one macrophage and lower instances of single cells. This phenotype was even more pronounced with the *fgr41Δ/Δ* mutant; up to nine *C. albicans* cells could be found in one macrophage, which was also significantly more than wild type, and single cells were rare. However, no significant differences were found between *fgr41Δ/Δ, fgr41Δ/Δ ENG1^oe^*, and *eng1Δ/Δfgr41Δ/Δ* cells (Fig. 2C). These results indicate that large clumps of cells are indeed being engulfed, and the increase in clump size correlates well with the TNF-α release induced by wild-type, *eng1Δ/Δ,* and *fgr41Δ/Δ* strains. However, the *fgr41∆/∆ ENG1^oe^* strain shows a significant decrease in TNF-α release from macrophages compared to the *fgr41Δ/Δ* strain (Fig. 1D), but the sizes of the clumps engulfed do not change (Fig. 2B through D), suggesting that clumping alone does not fully explain the differences in TNF-α.

### Macrophage stimulation by the *fgr41Δ/Δ* mutant is independent of dectin-1

**Fig 3:**
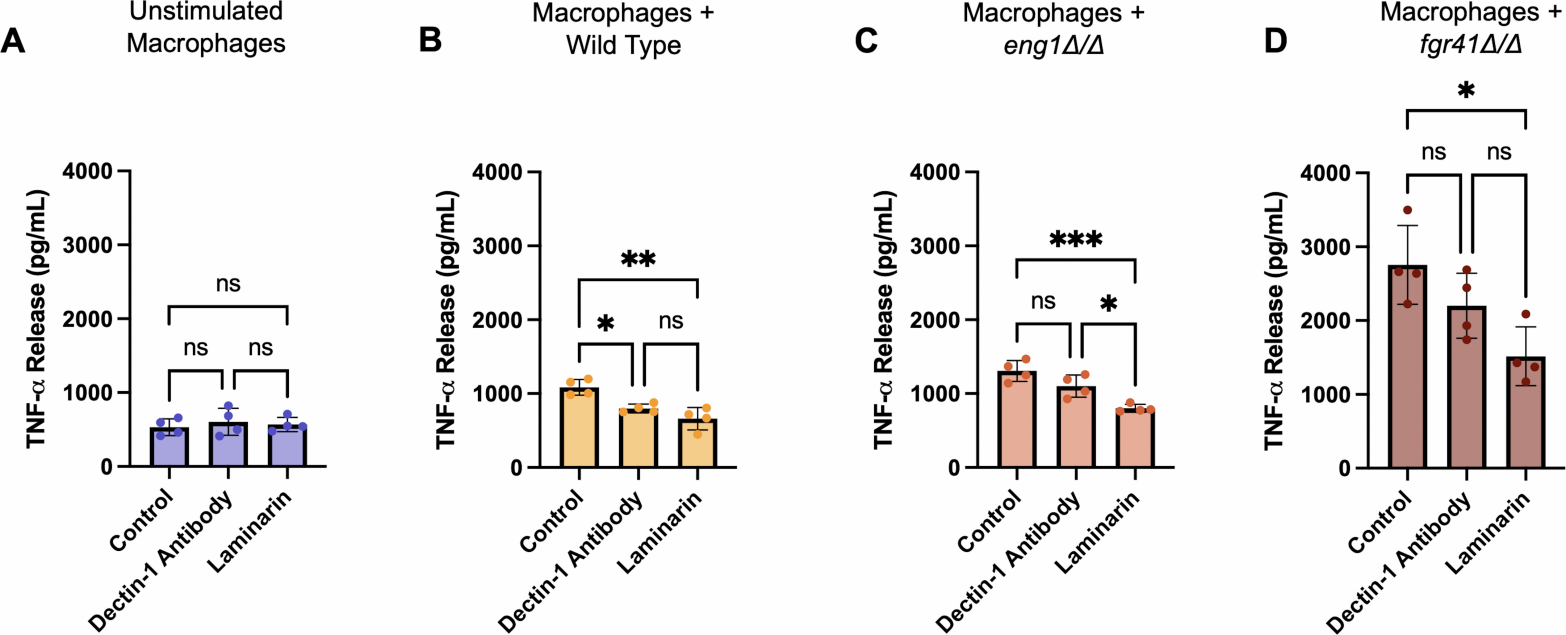
Effect of a neutralizing dectin-1 antibody or inhibitory laminarin on TNF-α release from macrophages stimulated by *eng1Δ/Δ* and *fgr41Δ/Δ* mutants. RAW 264.7 macrophages were treated with dectin-1 antibody (100 ng/mL) or inhibitory laminarin (100 µg/mL) for 1 h prior to a 4 h co-incubation with UV-inactivated *C. albicans* strains. TNF-α release was measured using an ELISA. Each experiment was performed four times with three replicates each time; each point represents the average of three technical replicates. (**A**) Unchallenged macrophages show no differences in TNF-α release when pretreated with dectin-1 antibody or laminarin. ns = not significant. (**B**) Wild type-challenged macrophages show a small decrease in TNF-α release after both dectin-1 antibody and laminarin pretreatment. **P < 0.01, *P < 0.05, by one-way ANOVA, ns = not significant. (**C**) *eng1Δ/Δ*-challenged macrophages exhibit a small but insignificant decrease in TNF-α release after pretreatment with dectin-1 antibody, and a significant decrease after pretreatment with laminarin. ***P < 0.001, *P < 0.05, by one-way ANOVA, ns = not significant. (**D**) Pretreatment with dectin-1 antibody did not significantly impact TNF-α release from macrophages incubated with *fgr41Δ/Δ,* although a small decrease was observed. Pretreatment with laminarin caused a large, significant reduction. *P < 0.05 by one-way ANOVA, ns = not significant.

It has been previously shown that blockage of dectin-1 leads to a significant decrease in recognition of *C. albicans* cells with high levels of exposed β(1, 3)-glucan (28). Additionally, small, soluble forms of β-glucan such as laminarin have been shown, in some cases, to inhibit β(1, 3)-glucan recognition in macrophages (48). In order to test the impact of dectin-1 recognition on the TNF-α release induced by the *eng1Δ/Δ* and *fgr41Δ/Δ* mutants, we treated RAW 264.7 macrophages with a neutralizing anti-dectin-1 antibody, an inhibitory form of laminarin, or PBS for 1 h prior to co-incubation. While unchallenged macrophages showed no differences between treatments, those challenged with the wild-type strain released significantly lower levels of TNF-α when pre-treated with either dectin-1 antibody or laminarin; however, there was not a significant difference between the levels of reduction caused by the two treatments (Fig. 3A and B). Challenge with the *eng1Δ/Δ* strain again caused increased levels of TNF-α release, which were reduced by both dectin-1 antibody and laminarin. However, laminarin caused a greater reduction, while the decrease due to dectin-1 antibody was insignificant (Fig. 3C). Similarly, the TNF-α levels induced by the *fgr41Δ/Δ* mutant in macrophages pre-treated with dectin-1 antibody were slightly lower than those of macrophages that received no antibody, but the difference was not significant (Fig. 3D). Laminarin pre-treatment, however, significantly reduced *fgr41Δ/Δ*-induced TNF-α levels by nearly half. Since the dectin-1 antibody inhibits only dectin-1, and laminarin should block all β-glucan recognition, this suggests that another macrophage receptor may be recognizing exposed β(1, 3)-glucan on the *fgr41Δ/Δ* and *eng1Δ/Δ* mutants.

### CR3 plays little to no role in immune stimulation by *fgr41Δ/Δ*

Another receptor expressed on macrophages that is known to recognize glucans is integrin α_M_β_2_, also known as Mac-1 or complement receptor 3 (CR3). CR3 is a heterodimeric β_2_-integrin receptor that binds a variety of different ligands. The α subunit of CR3, called CD11b, contains a binding site for microbial polysaccharides, including β-glucan (49). It has been shown that CR3 is important for neutrophil recognition of fungal β-glucan (50). Additionally, it has been implicated in macrophage binding and phagocytosis of zymosan, a form of β-glucan derived from the cell wall of *Saccharomyces cerevisiae* (51). To determine if this receptor is playing a role in recognition of the *fgr41Δ/Δ* mutant*,* macrophages were treated with a neutralizing antibody to CD11b for 1 h prior to co-incubation with wild-type *C. albicans* or the *fgr41Δ/Δ* strain. The antibody appeared to have no effect on the recognition of either strain (Fig. S3). This suggests that CR3 is not responsible for TNF-α induction occurring independent of dectin-1 recognition.

### Macrophage p38 but not NF-κB is activated upon stimulation with the *eng1Δ/Δ* or *fgr41Δ/Δ* strains

Stimulation of TNF-α production in macrophages can occur through many different signaling pathways, and the pathway that is stimulated can depend on the receptor used to recognize the ligand, as well as the ligand itself (52). In order to confirm the dectin-1 independence of *fgr41Δ/Δ*-induced stimulation and understand the signaling dynamics occurring upon recognition, we extracted protein from RAW 264.7 macrophages after co-incubation with each strain. Co-incubations were either performed for 30 min, in order to capture the early macrophage response, or for 4 h, to coincide with the time point at which supernatant was taken for analysis of TNF-α levels in previous experiments. We then performed western blots for common immune signaling molecules. A stimulatory form of laminarin, which induced high levels of TNF-α production from the macrophages, was employed as a positive control (48).

**Fig 4:**
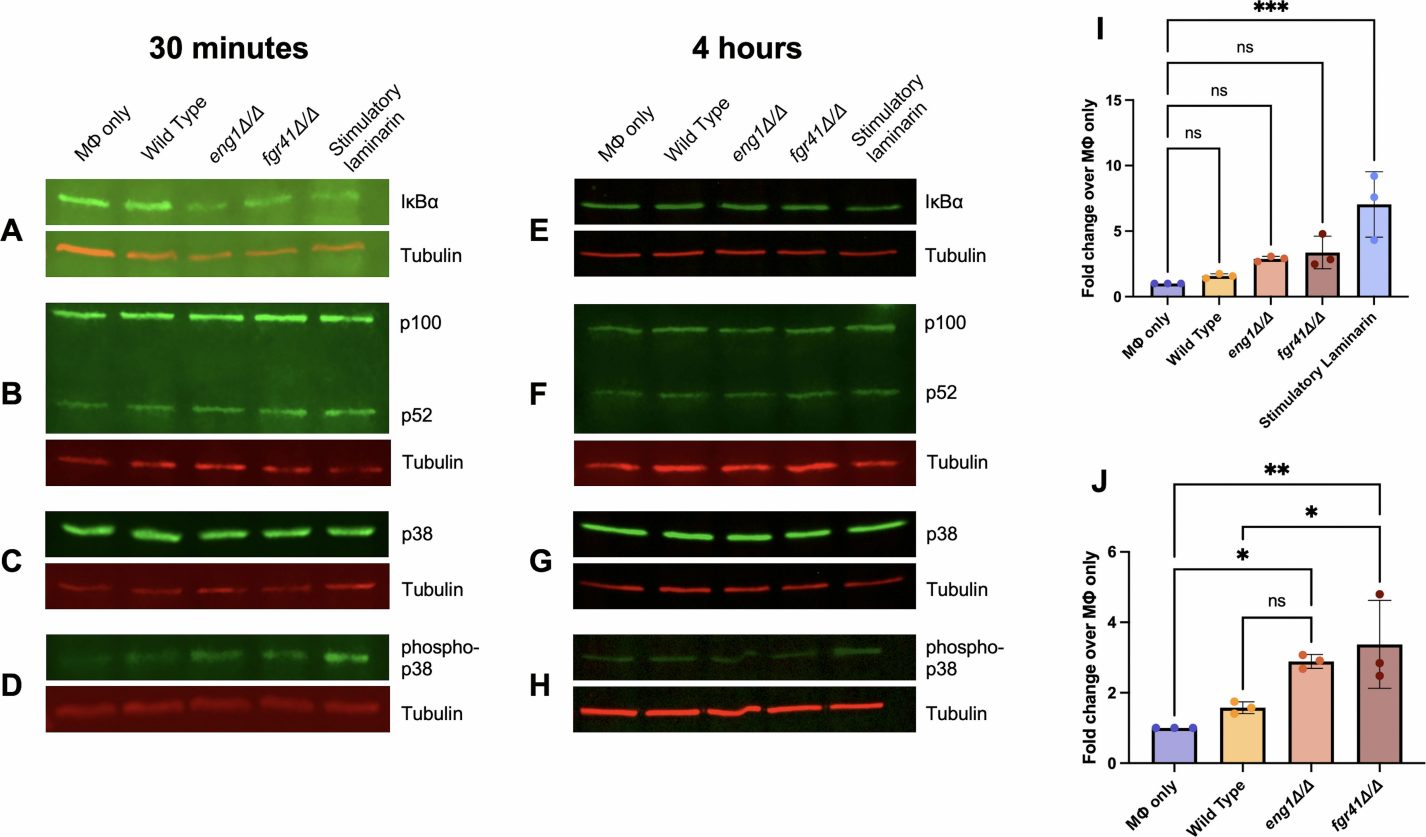
NF-κB and p38 activation stimulated by *eng1Δ/Δ* and *fgr41Δ/Δ* strains. Macrophages and *C. albicans* strains were co-incubated for 30 min (**A–D, I and J**) or 4 h (**E–H**). Macrophage protein was extracted and run on a 12% SDS-PAGE gel, and gels were transferred to PVDF membranes for immunoblotting. All membranes were blotted for tubulin as a loading control, with the primary antibody used at 1:1,000. (**A, E**) Membranes were blotted for IκBα, and 50 μg of protein was loaded into each well. The primary antibody to IκBα was used at 1:1,000. (**B, F**) Membranes were blotted for p100/p52, and 20 μg of protein was loaded into each well. The primary antibody was used at 1:1,000. (**C, G**) Membranes were blotted for p38, and 50 μg of protein was loaded into each well. The primary antibody to p38 was used at 1:1,000. (**D, H**) Membranes were blotted for phosphorylated p38, and 100 μg of protein was loaded into each well. The primary antibody was used at 1:500. (**I**) Quantification of D, including the positive control (stimulatory laminarin). Levels of phospho-p38 are significantly increased in the positive control condition. ***P < 0.001 by one-way ANOVA, ns = not significant. (**J**) Quantification of D, excluding the positive control condition. Both *eng1Δ/Δ-* and *fgr41Δ/Δ*-challenged macrophages show a significant increase in the production of phospho-p38 over untreated macrophages (MΦ only). Challenge with the *fgr41Δ/Δ* mutant also causes significantly more phospho-p38 production than challenge with wild-type *C. albicans*. **P <0.01, *P < 0.05, compared with wild-type (WT) by one-way ANOVA, ns = not significant.

The most well-studied transcription factor known to induce TNF-α production is NF-κB. It is a highly pleiotropic mediator of inflammation, and different dimeric combinations of NF-κB subunits can be formed by different signals (53). To capture the entirety of NF-κB activation, we first looked at levels of IκBα, an inhibitor of canonical NF-κB activation. When inactive, NF-κB dimers are bound to IκBα; to activate NF-κB, IκBα is phosphorylated, polyubiquitinated, and degraded (53). Therefore, higher levels of NF-κB activation should correlate with lower levels of IκBα protein. After 30 min of co-incubation, IκBα levels in the tested strains were highly variable between replicates, with no significant differences (Fig. 4A; Table S1). Furthermore, at 4 h, we did not observe any difference in IκBα protein levels between any of the tested conditions (Fig. 4E; Table S2).

Next, to look at non-canonical NF-κB activation, we measured levels of p100 and p52. Upon stimulation of the non-canonical pathway, the NF-κB precursor p100 is phosphorylated. This induces processing of the protein into the mature NF-κB subunit p52, which can then dimerize with another NF-κB subunit, RelB, and alter the transcription of a variety of genes (54). We observed increased production of p100 and p52 in the laminarin positive control after 30 min (Fig. 4B; Table S1) and increased p52 in that condition after 4 h (Fig. 4F; Table S2). However, no differences were observed between strains at either time point (Table S2).

Several different signaling pathways are activated during dectin-1 signaling, and the signaling molecules involved often crosstalk with other receptors (27). One major signaling molecule that acts downstream of dectin-1 but upstream of NF-κB is p38, a MAP kinase that is frequently activated during β(1, 3)-glucan recognition (55). To determine if this kinase was activated by our mutants, we blotted our macrophage protein extracts for unphosphorylated (inactive) and phosphorylated (active) p38. Total p38 levels were consistent across treatments, regardless of timing (Fig. 4C and G). Levels of active p38 (phospho-p38), however, were increased at 30 min in not only the positive control but the *eng1Δ/Δ* and *fgr41Δ/Δ* conditions as well (Fig. 4D, I and J). Most relevant, challenging macrophages with the *fgr41Δ/Δ* mutant caused significantly higher p38 activation than challenge with wild type (Fig. 4J). However, no strong differences besides the positive control are present at 4 h (Fig. 4H). Overall, this suggests that NF-κB is not playing a role in causing the TNF-α release stimulated by the *eng1Δ/Δ* and *fgr41Δ/Δ* strains, while p38 plays a role, at least in the *fgr41∆/∆* mutant, especially at earlier time points.

### The *fgr41Δ/Δ* mutant exhibits severely attenuated virulence in wild-type and *clec7a^-/-^* mice

A previous experiment demonstrating reduced kidney fungal burden in *fgr41Δ/Δ*-infected mice was performed using male outbred ICR mice (39). We wanted to determine if loss of *FGR41* would impact virulence in mice in a manner independent of dectin-1; however, this required the use of the inbred mouse line C57BL/6J, in which the dectin-1^-/-^ (*clec7a^-/-^*) mutation is found. To investigate the impact of the *fgr41Δ/Δ* mutation on virulence in C57BL/6J, we first measured kidney fungal burden. Mice were intravenously injected with 2.5 × 10^6^ *C. albicans* cells, either wild type or *fgr41Δ/Δ,* and the kidneys were plated for CFUs at 2 dpi. We found that the *fgr41Δ/Δ* mutant demonstrated reduced kidney colonization in both male and female C57BL/6J mice (Fig. S4).

**Fig 5:**
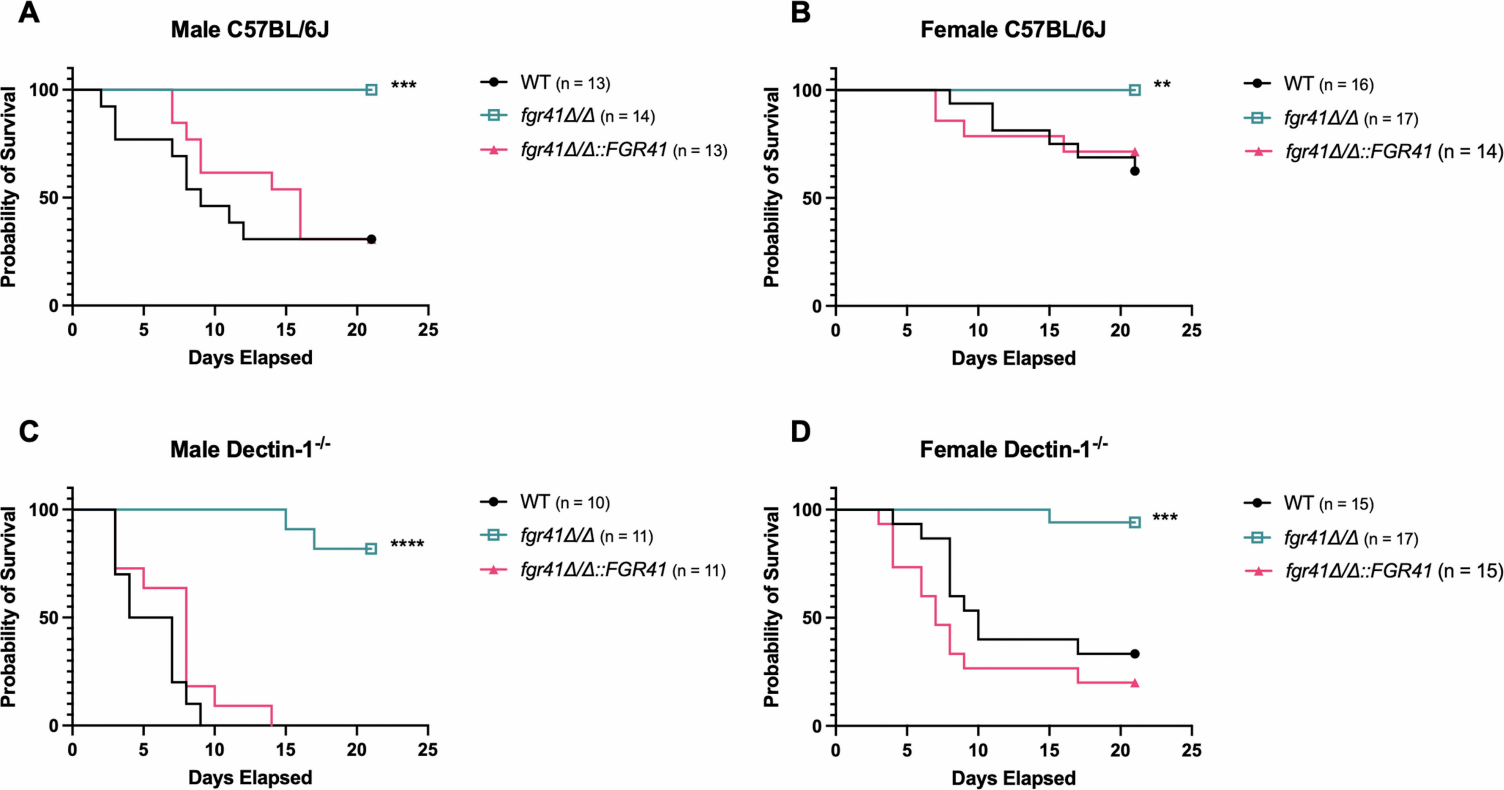
The *fgr41Δ/Δ* mutant results in decreased virulence in C57BL/6J and Dectin-1-/- mice. Mice were injected intravenously with 1 × 10^5^ cells of wild-type *C. albicans,* the *fgr41Δ/Δ* mutant*,* or the *FGR41* complement strain (*fgr41Δ/Δ::FGR41*). Survival was monitored over the course of 21 days. Each graph represents the combination of two replicate experiments, where *n* = the total number of mice infected with each strain across both experiments. (**A**) male and (**B**) female C57BL/6J mice infected with the *fgr41Δ/Δ* mutant showed complete survival over the course of the experiment, while those infected with wild-type or the complement strain showed significantly lower survival (***P < 0.001, **P < 0.01, compared to WT by Gehan-Breslow-Wilcoxon test). Similar trends were seen in (**C**) male and (**D**) female dectin-1 knockout mice. While mice infected with the *fgr41Δ/Δ* strains experienced limited mortality, they still survived significantly better than the wild-type- or complement-infected mice (****P < 0.0001, ***P < 0.001, compared to WT by Gehan Breslow-Wilcoxon test).

Having established a similar trend in fungal burden in these mice, we performed survival curves comparing the wild type and the *fgr41Δ/Δ* mutant in both wild-type C57BL/6J and dectin-1 knockout (*clec7a^-/-^*) mice. Male and female mice were intravenously injected with 1 × 10^5^ cells of either wild type, *fgr41Δ/Δ*, or *fgr41Δ/Δ::FGR41,* and survival was monitored over the course of 21 days. In both male (Fig. 5A) and female (Fig. 5B) C57BL/6J mice, infection with wild-type *C. albicans* or the *FGR41* complement resulted in significant mortality. However, mice infected with the *fgr41Δ/Δ* strain had 100% survival in both sexes. Dectin-1 knockout mice infected with wild type or *fgr41Δ/Δ::FGR41* strains showed reduced survival rates when compared to C57BL/6J mice infected with the same strain (Fig. 5C and D). Survival after infection with the *fgr41Δ/Δ* strain decreased slightly in dectin-1 knockout mice when compared with C57BL/6J, but virulence was still severly attenuated compared to the wild type and complement strains. Additionally, although dectin-1 knockout mice infected with the *fgr41Δ/Δ* mutant show a modest increase in mortality over C57BL/6J, the same holds true for wild-type and *fgr41Δ/Δ* complement strains, suggesting that the immune response to the *fgr41Δ/Δ* mutant *in vivo* is also independent of dectin-1.

## DISCUSSION

Previous work on both *FGR41* and *ENG1* strains has shown that knockouts of these genes share a preponderance of similar phenotypes. Both the *eng1Δ/Δ* and *fgr41Δ/Δ* mutants have increased β(1, 3)-glucan exposure concentrated at sites of active or former cell separation, as well as a defect in separation (37, 39). They are also both downregulated in unmasking conditions, including caspofungin treatment and *STE11* hyperactivation (37, 40, 41). Here, we have provided evidence that these proteins may work together in an overlapping manner to regulate β(1, 3)-glucan exposure and immune recognition in *C. albicans*. Overexpression of *ENG1* in the *fgr41Δ/Δ* background suppresses the high levels of β(1, 3)-glucan exposure caused by loss of *FGR41,* such that it is similar to wild type (Fig. 1A and B). Furthermore, TNF-α release induced from macrophages challenged with the *fgr41Δ/ΔENG1^oe^* mutant was similar to that of the *eng1Δ/Δ* mutant alone and was significantly lower than the very high levels of TNF-α secretion induced by the *fgr41Δ/Δ* mutant (Fig. 1D). Thus, overexpressed ENG1 can at least partially suppress both of these *fgr41Δ/Δ*-induced phenotypes. Eng1 is a secreted cell wall protein, and Fgr41 is predicted to be extracellularly localized as well; hence, it is possible that these two proteins are interacting at the cell surface (34, 38).

The unmasking of the *eng1Δ/Δfgr41Δ/Δ* double mutant provides further clues to the possible interaction between the two proteins. The double mutant showed less β(1, 3)-glucan exposure than the *eng1Δ/Δ* mutant but more than the *fgr41Δ/Δ* mutant (Fig. 1B). This may be due to the increased severity of the cell separation defect compared to the *eng1∆/∆* mutant; although the loss of Eng1 leads to unmasking at bud scars or growth areas, the stronger cell separation defect from the *fgr41Δ/Δ* mutation may protect some of the unmasked areas that would be exposed on *eng1Δ/Δ* individual cells. It is not clear what the function of Fgr41 is in the cell, but the fact that Eng1 overexpression can suppress the *fgr41Δ/Δ* unmasking phenotype, while Fgr41 overexpression does not suppress *eng1Δ/Δ* unmasking, suggests that Fgr41 may help coordinate other proteins for cell separation, including Eng1.

### β(1,3)-glucan exposure vs. immune response modulation by Fgr41 and Eng1

Our data indicate that there is no clear dose-dependent relationship between β(1, 3)-glucan exposure of our mutants and the TNF-α release they induce. Loss of *ENG1* caused a larger increase in β(1, 3)-glucan exposure than loss of *FGR41* (Fig. 1B), while loss of *FGR41* caused cells to stimulate more TNF-α from macrophages than loss of *ENG1* (Fig. 1D). The *eng1Δ/Δfgr41Δ/Δ* mutant was similar to the *fgr41Δ/Δ* mutant in TNF-α release, despite exhibiting lower β(1, 3)-glucan exposure than the *eng1∆/∆* mutant and higher than the *fgr41∆/∆* mutant (Fig. 1).

We initially hypothesized that the unmasking and TNF-α stimulation phenotypes we observed were due, in part, to changes in cell separation based on the phenotype seen in the *fgr41Δ/Δ* mutant. *FGR41* and *ENG1* are also known to be in the regulon of Ace2, a transcription factor that controls cell separation (56). However, although overexpression of *ENG1* in the *fgr41Δ/Δ* mutant caused a decrease in TNF-α release and β(1, 3)-glucan exposure, there was no change in cell separation. We also did not observe any major differences in cell separation or macrophage phagocytosis between the *fgr41Δ/Δ, fgr41Δ/Δ ENG1^oe^*, and *eng1Δ/Δfgr41Δ/Δ* strains (Fig. 2; Fig. S2). This suggests that while the formation of large clumps of cells does lead to more cells being phagocytosed, as these mutants were both clumpier than wild type, it does not explain the decrease in unmasking and TNF-α release induced when *ENG1* is overexpressed in the *fgr41Δ/Δ* mutant. It is possible that the β-glucanase activity of Eng1 in this strain may be able to break down β(1, 3)-glucan on the surface enough to reduce unmasking, but not enough to fully separate the cells. Alternatively, Eng1 may be mislocalized or improperly anchored in the absence of Fgr41, preventing it from acting properly on cell separation. In contrast, both *eng1Δ/Δ* and *fgr41Δ/Δ* were indeed phagocytosed in larger cell clumps than wild type, which could play a role in the immune stimulation they induce. Phagocytosis of such large clumps could be mediated by alternative processes such as LC3-associated phagocytosis (LAP), which employs autophagy machinery to augment cargo degradation and has been implicated in the response to *C. albicans* (57, 58). Nevertheless, *ENG1* overexpression in the *fgr41Δ/Δ* mutant did decrease TNF-α secretion but did not reduce the clumping, indicating that the increased TNF-α secretion is not solely a product of clumped cells (Fig. 1D and 2). These results collectively suggest that both the clumping and β(1, 3)-glucan exposure each make contributions to TNF-α secretion and that this may explain the lack of a clear dose-dependent relationship between β(1, 3)-glucan exposure and TNF-α release.

### Dectin-1-independent recognition and signaling in response to the *fgr41Δ/Δ* mutant

**Fig 6:**
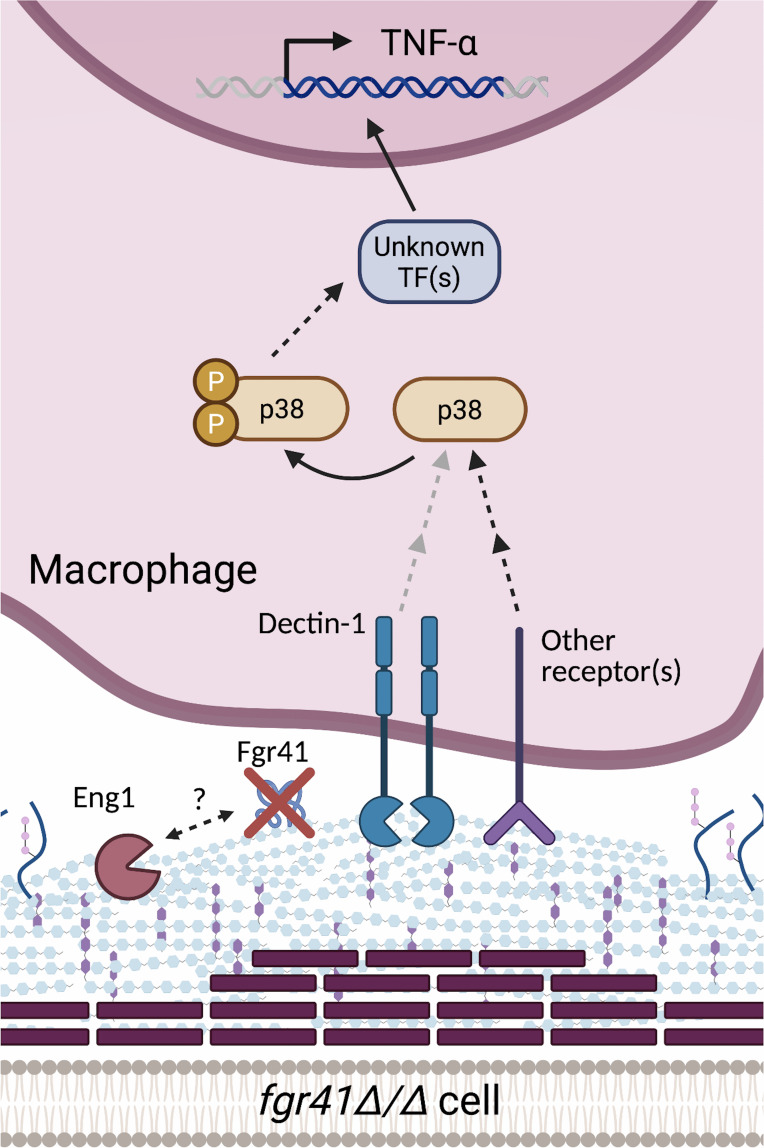
Proposed model. Eng1 localizes to bud necks and scars and degrades β(1, 3)-glucan at the septum during cell division. Eng1 may also be interacting with Fgr41 at the cell surface and can partially compensate for the loss of Fgr41 in unmasking and TNF-α stimulation. While exposed β(1, 3)-glucan in a wild-type cell is recognized primarily by dectin-1 on the surface of macrophages, recognition of the *fgr41Δ/Δ* mutant appears to be dectin-1-independent and is likely driven primarily by other receptors. Once the macrophage has recognized the *fgr41Δ/Δ* mutant, levels of phosphorylated p38 increase, and by this or other components, the signal is transduced to a transcription factor other than NF-κB to lead to TNF-α production.

Another interesting phenotype we discovered was the dectin-1 independence of the *fgr41Δ/Δ* and *eng1Δ/Δ* responses (Fig. 6). Dectin-1 antibody neutralization caused a decrease in TNF-α release stimulated from macrophages challenged with wild type; however, while the TNF-α release dropped slightly, the antibody had no significant impact on *eng1Δ/Δ-* or *fgr41Δ/Δ*-induced stimulation. This contradicts the findings of Yang et al. (37), who found a large reduction in TNF-α release stimulated by their *eng1Δ/Δ* strain from Dectin-1^-/-^ BMDMs compared to WT BMDMs (37). This suggests that differences in the macrophage source, *C. albicans* strain background, or culture conditions may play a role in dectin-1 recognition of this strain. No prior data on dectin-1 recognition of the *fgr41Δ/Δ* strain have been reported. While it is possible that dectin-1 is more important in recognition of the *fgr41Δ/Δ* strain in different *in vitro* conditions, we also observed a dectin-1 independent loss of virulence *in vivo* (Fig. 5), suggesting that alternative methods of recognition are indeed relevant.

The reduction of TNF-α release by inhibitory laminarin suggests that β(1, 3)-glucan exposure is important for this response (Fig. 3), but it is currently unclear what other receptor(s) are playing a role in recognition. A dectin-1 independent β(1, 3)-glucan response is not completely unprecedented, as CR3 can also be a driver of β(1, 3)-glucan recognition, but CR3 was also dispensable for the *fgr41Δ/Δ* response (Fig. S3). This was not entirely surprising either, as the role of CR3 in β(1, 3)-glucan recognition is somewhat controversial. Some sources have found CR3 to play a role in recognition of *C. albicans* β(1, 3)-glucan by macrophages, while others indicate that it is important in neutrophils but dispensable in macrophages (51, 59–62). Ephrin type-A receptor 2 (EphA2) is another receptor important for β(1, 3)-glucan recognition, but it also seems to play little role in macrophages (63). Dectin-1 and CR3-independent recognition of β(1, 3)-glucan has been reported but resulted in an anti-inflammatory response rather than an inflammatory one (64). No additional β(1, 3)-glucan receptors have been demonstrated to play a large role in *C. albicans* recognition, indicating that this response may come from a receptor that has yet to be implicated in β(1, 3)-glucan recognition. However, it is also possible that no single receptor is driving this response. It has been shown that TLR2 and dectin-1 can work together to recognize *C. albicans* (15). This or other undiscovered interactions between receptors could be playing a role in recognition of these mutants.

Investigation into the signaling molecules downstream of dectin-1 provided further evidence of a small role for dectin-1, as there was no clear activation of canonical or non-canonical NF-κB signaling after 30 min or 4 h of co-incubation with our strains (Fig. 4). This seems unusual, as NF-κB is thought to be the main transcription factor driving TNF-α production (53). However, IκBα degradation has been found to occur just 10 min after stimulation with *C. albicans*, suggesting earlier time points may have yielded different results (65). p38 did appear to be activated, and the *fgr41∆/∆* mutant stimulated greater phosphorylation than wild type at the 30-min time point (Fig. 4D). While this provides a clue as to what receptors may be involved in recognition of the *fgr41∆/∆* mutant, p38 is a highly pleiotropic signaling molecule that can be activated by a large variety of receptors; hence, the method of recognition is still unclear (66).

In addition to the evidence gathered *in vitro*, we also show that survival after systemic infection with the *fgr41Δ/Δ* mutant appears to be independent of dectin-1 in a murine systemic infection model (Fig. 5). A slight drop in survival may be consistent with the nonsignificant but minor drop in TNF-α release upon *fgr41Δ/Δ* challenge *in vitro* (Fig. 3C). Dectin-1 may therefore play a small role in recognition, but it is clear that the immune response is primarily driven by another receptor. Survival in *fgr41Δ/Δ*-infected mice was also similar between males and females. However, we do see increased survival in females infected with wild type *C. albicans* compared to males, regardless of the presence of dectin-1. The reason behind this is unclear; a previous study demonstrated similar results, with females injected with wild type surviving better, but no explanation was found (37).

## MATERIALS AND METHODS

### Materials

RAW 264.7 macrophages were used in this study. Antibodies to p38 (9212S), phospho-p38 (9211S), IκBα (9242S), and p100/p52 (52583S) were purchased from Cell Signaling Technologies. The primary antibody to exposed β(1, 3)-glucan was purchased from BioSupplies Australia (400-2). Alexa Fluor 488 goat anti-mouse secondary antibody was purchased from Jackson Immunoresearch (115-545-003). Inhibitory laminarin was purchased from Invivogen (LMN-38-01). Stimulatory laminarin was purchased from Millipore Sigma (L9634). CellBrite Green stain was purchased from Biotium (30021). Mouse TNF-α ELISA kits were purchased from R&D Systems (DY410).

### Growth and culture conditions

Unless otherwise specified, all *C. albicans* strains were grown in 5 mL of yeast peptone dextrose (YPD) medium to stationary phase overnight at 30°C, shaking at 225 rpm. DH5-α *E. coli* strains used for cloning were grown in LB broth supplemented with 100 μg/mL ampicillin overnight at 37°C on a rotating wheel. RAW 264.7 macrophages were grown in Dulbecco's modified Eagle medium (DMEM) supplemented with 10% fetal bovine serum (FBS) and 1% penicillin/streptomycin and maintained at 37°C + 5% CO_2_.

### Strain construction

*C. albicans* strains used in this study are listed in Table S3. *FGR41* knockout, overexpression, and complement strains were created previously (39). The previous *FGR41* overexpression plasmid, pSL003, was used for all new strains requiring *FGR41* overexpression (Table S4). The *ENG1* overexpression strain (*ENG1^oe^*) was created by amplifying the *ENG1* open reading frame (ORF) with ~500 base pairs of the 3’ untranslated region (UTR) from wild-type genomic DNA with primers AKO1 and AKO2, which contain *Not*I restriction sites (Table S5). The product was then cloned into the plasmid pBT1 by restriction cloning at the *Not*I site. The resulting plasmid, pAEK012, was used for all strains requiring *ENG1* overexpression.

*ENG1* knockout strains were created using CRISPR-Cas9 as previously described (45). Repair templates were created by amplifying regions of plasmids pBSS2 and CaHygB-flipper (containing either nourseothricin resistance or hygromycin resistance, respectively) using primers AKO57 and AKO58. Repair templates, along with ribonucleoprotein (RNP) complexes containing Cas9 and guide RNAs specific for *ENG1*, were then transformed into *C. albicans* to excise the gene. Successful deletion was confirmed by PCR using primers AWO413 and AWO414, as well as growth on YPD containing 200 µg/mL nourseothricin and 600 µg/mL hygromycin.

### Staining for confocal microscopy

To stain for exposed β(1, 3)-glucan and total chitin, *C. albicans* strains were grown overnight and back-diluted to an OD_600_ of 0.5 in PBS. Cells were blocked in 3% bovine serum albumin (BSA) for 30 min prior to staining and then incubated with a primary antibody to β(1, 3)-glucan at a 1:800 dilution in 3% BSA for 1.5 h. After washing, a goat anti-mouse secondary antibody conjugated to Alexa Fluor 488 was added at a 1:300 dilution in 5% BSA for 20 min. Cells were then incubated in 0.01 mg/mL calcofluor white (CFW) for 5 min at room temperature in the dark before being washed and imaged. Image analysis was performed using Fiji ImageJ.

### Macrophage-*Candida* co-incubations

To measure TNF-α release from RAW 264.7 cells, macrophages were seeded in a 24-well plate at 5 × 10^5^ cells/well and allowed to adhere overnight at 37°C + 5% CO_2_. To inhibit macrophage receptor recognition, macrophages were treated with indicated concentrations of dectin-1 antibody, CD11b antibody, inhibitory laminarin, or PBS for 1 hour at 37°C + 5% CO_2_ prior to the addition of *C. albicans. C. albicans* strains were grown overnight and diluted to an OD_600_ of 1.25 in 5 mL of PBS in a 6-well plate. They were then UV-inactivated, and 100 μL of cells was added to each well. Macrophages and *C. albicans* were co-incubated for 4 h at 37°C + 5% CO_2_ before the supernatant was removed, filtered, and frozen at −80°C. TNF-α concentrations were measured by enzyme-linked immunosorbent assay (ELISA) as previously described (67).

For confocal microscopy, 5 × 10^4^ macrophages were seeded in an 8-well chamber slide (CellTreat) and allowed to adhere overnight at 37°C + 5% CO_2_. *C. albicans* strains were UV-inactivated, and 1.25 × 10^5^ cells were added to each chamber. Macrophages and *C. albicans* were co-incubated for 2 h at 37°C + 5% CO_2_. The slides were then fixed with 4% formaldehyde for 30 min. Macrophages were stained with CellBrite Green at a 1:200 dilution for 20 min. After washing with PBS, the *C. albicans* cells were stained with 0.01 mg/mL CFW for 5 min. Cells were then washed and mounted in PBS before imaging. Image analysis was performed using Fiji ImageJ.

### Protein extraction and western blotting

To extract macrophage protein, 5 × 10^6^ macrophages were seeded in a T75 flask with 20 mL media and allowed to adhere overnight at 37°C + 5% CO_2_. *C. albicans* strains were grown overnight, diluted to an OD_600_ of 1.25 in 5 mL of PBS in a 6-well plate, and UV-inactivated; 1 mL of *C. albicans* cells, PBS, or 10 mg/mL stimulatory laminarin was added to each flask, and flasks were incubated for 30 min or 4 h at 37°C + 5% CO_2_. Macrophages were washed twice with cold PBS, then gently scraped off the bottom of the well into suspension in cold PBS. They were then spun down at 400 × *g* for 5 min and resuspended in cold RIPA buffer with protease and phosphatase inhibitors. Cells were allowed to incubate for 30 min at 4°C before being centrifuged at 12,000 × *g* for 10 min at 4°C. Protein concentrations in the resulting supernatant were quantified by Bradford assay or Pierce 660 protein concentration assay. Protein samples were then diluted to the indicated concentrations, run on SDS-PAGE, transferred to a PVDF membrane, and immunoblotted at the dilutions noted above.

### Systemic murine model of infection

Wild-type C57BL/6J and Dectin-1^-/-^ (Cle7a-/-) mice were purchased from The Jackson Laboratory (Bar Harbor, ME) (JAX stock #012337) (68) and bred under an Institutional Animal Care and Use Committee (IACUC)-approved protocol at The University of Tennessee, Knoxville.

To generate survival curves, *C. albicans* strains were grown overnight in 5 mL of YPD, shaking at 225 rpm at 30°C, then back-diluted the following morning into 50 mL fresh YPD and grown to mid-log phase, ~4 h. Cells were then washed twice with PBS and counted via hemacytometer to prepare a 1 × 10^6^ cells/mL dilution of each strain. Mice were then injected intravenously via the lateral tail vein with 100 μL of the prepared cell dilution. After injections, each strain was plated for viability on YPD and grown at 30°C for 48 h. Mice were then monitored for survival over the course of 21 days.

Kidney fungal burden was determined as described previously with slight modification (67). Cells were grown overnight, washed twice the following morning in PBS, then diluted to 2.5 × 10^7^ cells/mL. Mice were injected with 100 μL of cell suspension (which was also plated for viability), and kidneys were harvested 2 days post-infection. Kidneys were weighed, homogenized, and serially diluted in PBS and plated on YPD + 75 µg/mL chloramphenicol. Plates were then incubated for 48 h at 30°C and then counted for colony-forming units (CFUs).

## SUPPLEMENTAL MATERIAL

Supplemental material iai.00523-25-s0001.pdf Fig. S1 to S4; Tables S1 to S5.

ASM does not own the copyrights to Supplemental Material that may be linked to, or accessed through, an article. The authors have granted ASM a non-exclusive, world-wide license to publish the Supplemental Material files. Please contact the corresponding author directly for reuse.

## References

[1] Kashyap B, Padala SR, Kaur G, Kullaa A. 2024. Candida albicans induces oral microbial dysbiosis and promotes oral diseases. Microorganisms 12:2138. doi:10.3390/microorganisms12112138 39597528 PMC11596246

[2] Bhosale VB, Koparde AA, Thorat VM. 2025. Vulvovaginal candidiasis-an overview of current trends and the latest treatment strategies. Microb Pathog 200:107359. doi:10.1016/j.micpath.2025.107359 39921042

[3] Barantsevich N, Barantsevich E. 2022. Diagnosis and treatment of invasive candidiasis. Antibiotics (Basel) 11:718. doi:10.3390/antibiotics11060718 35740125 PMC9219674

[4] Netea MG, Brown GD, Kullberg BJ, Gow NAR. 2008. An integrated model of the recognition of Candida albicans by the innate immune system. Nat Rev Microbiol 6:67–78. doi:10.1038/nrmicro1815 18079743

[5] Brown GD, Denning DW, Gow NAR, Levitz SM, Netea MG, White TC. 2012. Hidden killers: human fungal infections. Sci Transl Med 4:165rv13. doi:10.1126/scitranslmed.3004404 23253612

[6] Pfaller MA, Diekema DJ. 2007. Epidemiology of invasive candidiasis: a persistent public health problem. Clin Microbiol Rev 20:133–163. doi:10.1128/CMR.00029-06 17223626 PMC1797637

[7] Cornely OA, Sprute R, Bassetti M, Chen SC-A, Groll AH, Kurzai O, Lass-Flörl C, Ostrosky-Zeichner L, Rautemaa-Richardson R, Revathi G, et al.. 2025. Global guideline for the diagnosis and management of candidiasis: an initiative of the ECMM in cooperation with ISHAM and ASM. Lancet Infect Dis 25:e280–e293. doi:10.1016/S1473-3099(24)00749-7 39956121

[8] Czajka KM, Venkataraman K, Brabant-Kirwan D, Santi SA, Verschoor C, Appanna VD, Singh R, Saunders DP, Tharmalingam S. 2023. Molecular mechanisms associated with antifungal resistance in pathogenic Candida species. Cells 12:2655. doi:10.3390/cells12222655 37998390 PMC10670235

[9] Davidson L, Netea MG, Kullberg BJ. 2018. Patient susceptibility to candidiasis-a potential for adjunctive Immunotherapy. J Fungi (Basel) 4:9. doi:10.3390/jof4010009 29371502 PMC5872312

[10] Bojang E, Ghuman H, Kumwenda P, Hall RA. 2021. Immune sensing of Candida albicans. JoF 7:119. doi:10.3390/jof7020119 33562068 PMC7914548

[11] Hall RA, Gow NAR. 2013. Mannosylation in Candida albicans: role in cell wall function and immune recognition. Mol Microbiol 90:1147–1161. doi:10.1111/mmi.12426 24125554 PMC4112839

[12] Swidergall M. 2019. Candida albicans at host barrier sites: pattern recognition receptors and beyond. Pathogens 8:40. doi:10.3390/pathogens8010040 30934602 PMC6471378

[13] Zheng NX, Wang Y, Hu DD, Yan L, Jiang YY. 2015. The role of pattern recognition receptors in the innate recognition of Candida albicans. Virulence 6:347–361. doi:10.1080/21505594.2015.1014270 25714264 PMC4601294

[14] Jouault T, Ibata-Ombetta S, Takeuchi O, Trinel PA, Sacchetti P, Lefebvre P, Akira S, Poulain D. 2003. Candida albicans phospholipomannan is sensed through toll-like receptors. J Infect Dis 188:165–172. doi:10.1086/375784 12825186

[15] Netea MG, Gow NAR, Munro CA, Bates S, Collins C, Ferwerda G, Hobson RP, Bertram G, Hughes HB, Jansen T, Jacobs L, Buurman ET, Gijzen K, Williams DL, Torensma R, McKinnon A, MacCallum DM, Odds FC, Van der Meer JWM, Brown AJP, Kullberg BJ. 2006. Immune sensing of Candida albicans requires cooperative recognition of mannans and glucans by lectin and toll-like receptors. J Clin Invest 116:1642–1650. doi:10.1172/JCI27114 16710478 PMC1462942

[16] Sancho D, Reis e Sousa C. 2012. Signaling by myeloid C-type lectin receptors in immunity and homeostasis. Annu Rev Immunol 30:491–529. doi:10.1146/annurev-immunol-031210-101352 22224766 PMC4480235

[17] Wheeler RT, Fink GR. 2006. A drug-sensitive genetic network masks fungi from the immune system. PLoS Pathog 2:e35. doi:10.1371/journal.ppat.0020035 16652171 PMC1447670

[18] Graus MS, Wester MJ, Lowman DW, Williams DL, Kruppa MD, Martinez CM, Young JM, Pappas HC, Lidke KA, Neumann AK. 2018. Mannan molecular substructures control nanoscale glucan exposure in Candida. Cell Rep 24:2432–2442. doi:10.1016/j.celrep.2018.07.088 30157435 PMC6204226

[19] Bain JM, Louw J, Lewis LE, Okai B, Walls CA, Ballou ER, Walker LA, Reid D, Munro CA, Brown AJP, Brown GD, Gow NAR, Erwig LP. 2014. Candida albicans hypha formation and mannan masking of β-glucan inhibit macrophage phagosome maturation. mBio 5:e01874. doi:10.1128/mBio.01874-14 25467440 PMC4324242

[20] Chen T, Wagner AS, Reynolds TB. 2022. When is it appropriate to take off the mask? Signaling pathways that regulate ß(1,3)-glucan exposure in Candida albicans. Front Fungal Biol 3:842501. doi:10.3389/ffunb.2022.842501 36908584 PMC10003681

[21] Childers DS, Avelar GM, Bain JM, Pradhan A, Larcombe DE, Netea MG, Erwig LP, Gow NAR, Brown AJP. 2020. Epitope shaving promotes fungal immune evasion. mBio 11:e00984-20. doi:10.1128/mBio.00984-20 32636248 PMC7343991

[22] Cottier F, Sherrington S, Cockerill S, Del Olmo Toledo V, Kissane S, Tournu H, Orsini L, Palmer GE, Pérez JC, Hall RA. 2019. Remasking of Candida albicans β-glucan in response to environmental ph is regulated by quorum sensing. mBio 10:e02347-19. doi:10.1128/mBio.02347-19 31615961 PMC6794483

[23] Guirao-Abad JP, Sánchez-Fresneda R, Machado F, Argüelles JC, Martínez-Esparza M. 2018. Micafungin enhances the human macrophage response to Candida albicans through β-glucan exposure. Antimicrob Agents Chemother 62:e02161-17. doi:10.1128/AAC.02161-17 29483123 PMC5923102

[24] de Assis LJ, Bain JM, Liddle C, Leaves I, Hacker C, Peres da Silva R, Yuecel R, Bebes A, Stead D, Childers DS, Pradhan A, Mackenzie K, Lagree K, Larcombe DE, Ma Q, Avelar GM, Netea MG, Erwig LP, Mitchell AP, Brown GD, Gow NAR, Brown AJP. 2022. Nature of β-1,3-glucan-exposing features on Candida albicans cell wall and their modulation. mBio 13:e0260522. doi:10.1128/mbio.02605-22 36218369 PMC9765427

[25] Ballou ER, Avelar GM, Childers DS, Mackie J, Bain JM, Wagener J, Kastora SL, Panea MD, Hardison SE, Walker LA, Erwig LP, Munro CA, Gow NAR, Brown GD, MacCallum DM, Brown AJP. 2016. Lactate signalling regulates fungal β-glucan masking and immune evasion. Nat Microbiol 2:16238. doi:10.1038/nmicrobiol.2016.238 27941860 PMC5704895

[26] Wheeler RT, Kombe D, Agarwala SD, Fink GR. 2008. Dynamic, morphotype-specific Candida albicans beta-glucan exposure during infection and drug treatment. PLoS Pathog 4:e1000227. doi:10.1371/journal.ppat.1000227 19057660 PMC2587227

[27] Mata-Martínez P, Bergón-Gutiérrez M, Del Fresno C. 2022. Dectin-1 signaling update: new perspectives for trained immunity. Front Immunol 13:812148. doi:10.3389/fimmu.2022.812148 35237264 PMC8882614

[28] Gow NAR, Netea MG, Munro CA, Ferwerda G, Bates S, Mora-Montes HM, Walker L, Jansen T, Jacobs L, Tsoni V, Brown GD, Odds FC, Van der Meer JWM, Brown AJP, Kullberg BJ. 2007. Immune recognition of Candida albicans beta-glucan by dectin-1. J Infect Dis 196:1565–1571. doi:10.1086/523110 18008237 PMC2655640

[29] Halder LD, Jo EAH, Hasan MZ, Ferreira-Gomes M, Krüger T, Westermann M, Palme DI, Rambach G, Beyersdorf N, Speth C, Jacobsen ID, Kniemeyer O, Jungnickel B, Zipfel PF, Skerka C. 2020. Immune modulation by complement receptor 3-dependent human monocyte TGF-β1-transporting vesicles. Nat Commun 11:2331. doi:10.1038/s41467-020-16241-5 32393780 PMC7214408

[30] Li X, Utomo A, Cullere X, Choi MM, Milner DA, Venkatesh D, Yun SH, Mayadas TN. 2011. The β-glucan receptor dectin-1 activates the integrin Mac-1 in neutrophils via Vav protein signaling to promote Candida albicans clearance. Cell Host Microbe 10:603–615. doi:10.1016/j.chom.2011.10.009 22177564 PMC3244687

[31] Zhang SQ, Zou Z, Shen H, Shen SS, Miao Q, Huang X, Liu W, Li LP, Chen SM, Yan L, Zhang JD, Zhao JJ, Xu GT, An MM, Jiang YY. 2016. Mnn10 maintains pathogenicity in Candida albicans by extending α-1,6-mannose backbone to evade host dectin-1 mediated antifungal immunity. PLoS Pathog 12:e1005617. doi:10.1371/journal.ppat.1005617 27144456 PMC4856274

[32] Davis SE, Hopke A, Minkin SC, Montedonico AE, Wheeler RT, Reynolds TB. 2014. Masking of β(1-3)-glucan in the cell wall of Candida albicans from detection by innate immune cells depends on phosphatidylserine. Infect Immun 82:4405–4413. doi:10.1128/IAI.01612-14 25114110 PMC4187869

[33] Baladrón V, Ufano S, Dueñas E, Martín-Cuadrado AB, del Rey F, Vázquez de Aldana CR. 2002. Eng1p, an endo-1,3-beta-glucanase localized at the daughter side of the septum, is involved in cell separation in Saccharomyces cerevisiae. Eukaryot Cell 1:774–786. doi:10.1128/EC.1.5.774-786.2002 12455695 PMC126745

[34] Esteban PF, Ríos I, García R, Dueñas E, Plá J, Sánchez M, de Aldana CRV, Del Rey F. 2005. Characterization of the CaENG1 gene encoding an endo-1,3-beta-glucanase involved in cell separation in Candida albicans. Curr Microbiol 51:385–392. doi:10.1007/s00284-005-0066-2 16328626

[35] Martín-Cuadrado AB, Dueñas E, Sipiczki M, Vázquez de Aldana CR, del Rey F. 2003. The endo-beta-1,3-glucanase eng1p is required for dissolution of the primary septum during cell separation in Schizosaccharomyces pombe. J Cell Sci 116:1689–1698. doi:10.1242/jcs.00377 12665550

[36] Garfoot AL, Shen Q, Wüthrich M, Klein BS, Rappleye CA. 2016. The Eng1 β-glucanase enhances histoplasma virulence by reducing β-glucan exposure. mBio 7:e01388-15. doi:10.1128/mBio.01388-15 27094334 PMC4850272

[37] Yang M, Solis NV, Marshall M, Garleb R, Zhou T, Wang D, Swidergall M, Pearlman E, Filler SG, Liu H. 2022. Control of β-glucan exposure by the endo-1,3-glucanase Eng1 in Candida albicans modulates virulence. PLoS Pathog 18:e1010192. doi:10.1371/journal.ppat.1010192 34995333 PMC8775328

[38] De Groot PWJ, Hellingwerf KJ, Klis FM. 2003. Genome-wide identification of fungal GPI proteins. Yeast 20:781–796. doi:10.1002/yea.1007 12845604

[39] Wagner AS, Lumsdaine SW, Mangrum MM, King AE, Hancock TJ, Sparer TE, Reynolds TB. 2022. Cek1 regulates ß(1,3)-glucan exposure through calcineurin effectors in Candida albicans. PLoS Genet 18:e1010405. doi:10.1371/journal.pgen.1010405 36121853 PMC9521907

[40] Chen T, Wagner AS, Tams RN, Eyer JE, Kauffman SJ, Gann ER, Fernandez EJ, Reynolds TB. 2019. Lrg1 regulates β (1,3)-glucan masking in Candida albicans through the Cek1 MAP kinase pathway. mBio 10:e01767-19. doi:10.1128/mBio.01767-19 31530671 PMC6751057

[41] Bruno VM, Kalachikov S, Subaran R, Nobile CJ, Kyratsous C, Mitchell AP. 2006. Control of the C. albicans cell wall damage response by transcriptional regulator Cas5. PLoS Pathog 2:e21. doi:10.1371/journal.ppat.0020021 16552442 PMC1401495

[42] Liu TT, Lee REB, Barker KS, Lee RE, Wei L, Homayouni R, Rogers PD. 2005. Genome-wide expression profiling of the response to azole, polyene, echinocandin, and pyrimidine antifungal agents in Candida albicans. Antimicrob Agents Chemother 49:2226–2236. doi:10.1128/AAC.49.6.2226-2236.2005 15917516 PMC1140538

[43] Chaudhuri R, Ansari FA, Raghunandanan MV, Ramachandran S. 2011. FungalRV: adhesin prediction and immunoinformatics portal for human fungal pathogens. BMC Genomics 12:192. doi:10.1186/1471-2164-12-192 21496229 PMC3224177

[44] Mercado Soto NM, Horn A, Keller NP, Huttenlocher A, Wagner AS. 2025. Larval zebrafish burn wound infection model reveals conserved innate immune responses against diverse pathogenic fungi. mBio 16:e0348024. doi:10.1128/mbio.03480-24 40197062 PMC12077223

[45] Liu J, Vogel AK, Miao J, Carnahan JA, Lowes DJ, Rybak JM, Peters BM. 2022. Rapid hypothesis testing in Candida albicans clinical isolates using a cloning-free, modular, and recyclable system for CRISPR-Cas9 mediated mutant and revertant construction. Microbiol Spectr 10:e0263021. doi:10.1128/spectrum.02630-21 35612314 PMC9241802

[46] Wagner AS, Vogel AK, Lumsdaine SW, Phillips EK, Willems HME, Peters BM, Reynolds TB. 2022. Mucosal infection with unmasked Candida albicans cells impacts disease progression in a host niche-specific manner. Infect Immun 90:e0034222. doi:10.1128/iai.00342-22 36374100 PMC9753624

[47] Oliver JC, Ferreira CBRJ, Silva NC, Dias ALT. 2019. Candida spp. and phagocytosis: multiple evasion mechanisms. Antonie Van Leeuwenhoek 112:1409–1423. doi:10.1007/s10482-019-01271-x 31079344

[48] Smith AJ, Graves B, Child R, Rice PJ, Ma Z, Lowman DW, Ensley HE, Ryter KT, Evans JT, Williams DL. 2018. Immunoregulatory activity of the natural product laminarin varies widely as a result of its physical properties. J Immunol 200:788–799. doi:10.4049/jimmunol.1701258 29246954 PMC5760317

[49] Lamers C, Plüss CJ, Ricklin D. 2021. The promiscuous profile of complement receptor 3 in ligand binding, immune modulation, and pathophysiology. Front Immunol 12:662164. doi:10.3389/fimmu.2021.662164 33995387 PMC8118671

[50] Lavigne LM, Albina JE, Reichner JS. 2006. Beta-glucan is a fungal determinant for adhesion-dependent human neutrophil functions. J Immunol 177:8667–8675. doi:10.4049/jimmunol.177.12.8667 17142767

[51] Hadas S, Reichert F, Rotshenker S. 2010. Dissimilar and similar functional properties of complement receptor-3 in microglia and macrophages in combating yeast pathogens by phagocytosis. Glia 58:823–830. doi:10.1002/glia.20966 20091776

[52] Pradervand S, Maurya MR, Subramaniam S. 2006. Identification of signaling components required for the prediction of cytokine release in RAW 264.7 macrophages. Genome Biol 7:R11. doi:10.1186/gb-2006-7-2-r11 16507166 PMC1431720

[53] Dorrington MG, Fraser IDC. 2019. NF-κB signaling in macrophages: dynamics, crosstalk, and signal integration. Front Immunol 10:705. doi:10.3389/fimmu.2019.00705 31024544 PMC6465568

[54] Sun SC. 2011. Non-canonical NF-κB signaling pathway. Cell Res 21:71–85. doi:10.1038/cr.2010.177 21173796 PMC3193406

[55] Alsina-Beauchamp D, Escós A, Fajardo P, González-Romero D, Díaz-Mora E, Risco A, Martín-Serrano MA, Del Fresno C, Dominguez-Andrés J, Aparicio N, Zur R, Shpiro N, Brown GD, Ardavín C, Netea MG, Alemany S, Sanz-Ezquerro JJ, Cuenda A. 2018. Myeloid cell deficiency of p38γ/p38δ protects against candidiasis and regulates antifungal immunity. EMBO Mol Med 10:e8485. doi:10.15252/emmm.201708485 29661910 PMC5938613

[56] Mulhern SM, Logue ME, Butler G. 2006. Candida albicans transcription factor Ace2 regulates metabolism and is required for filamentation in hypoxic conditions. Eukaryot Cell 5:2001–2013. doi:10.1128/EC.00155-06 16998073 PMC1694816

[57] Herb M, Gluschko A, Schramm M. 2020. LC3-associated phagocytosis - the highway to hell for phagocytosed microbes. Semin Cell Dev Biol 101:68–76. doi:10.1016/j.semcdb.2019.04.016 31029766

[58] Tam JM, Mansour MK, Acharya M, Sokolovska A, Timmons AK, Lacy-Hulbert A, Vyas JM. 2016. The role of autophagy-related proteins in Candida albicans infections. Pathogens 5:34. doi:10.3390/pathogens5020034 27043636 PMC4931385

[59] Brown GD, Taylor PR, Reid DM, Willment JA, Williams DL, Martinez-Pomares L, Wong SYC, Gordon S. 2002. Dectin-1 is a major beta-glucan receptor on macrophages. J Exp Med 196:407–412. doi:10.1084/jem.20020470 12163569 PMC2193936

[60] Taylor PR, Brown GD, Reid DM, Willment JA, Martinez-Pomares L, Gordon S, Wong SYC. 2002. The β-glucan receptor, dectin-1, is predominantly expressed on the surface of cells of the monocyte/macrophage and neutrophil lineages. J Immunol 169:3876–3882. doi:10.4049/jimmunol.169.7.3876 12244185

[61] Li D, Bai CS, Zhang Q, Li Z, Shao D, Li XC. 2019. β-1,3-Glucan/CR3/SYK pathway-dependent LC3B-II accumulation enhanced the fungicidal activity in human neutrophils. J Microbiol 57:263–270. doi:10.1007/s12275-019-8298-1 30721460

[62] Forsyth CB, Plow EF, Zhang L. 1998. Interaction of the fungal pathogen Candida albicans with integrin CD11b/CD18: recognition by the I domain is modulated by the lectin-like domain and the CD18 subunit. J Immunol 161:6198–6205. doi:10.4049/jimmunol.161.11.6198 9834106

[63] Swidergall M, Solis NV, Wang Z, Phan QT, Marshall ME, Lionakis MS, Pearlman E, Filler SG. 2019. EphA2 is a neutrophil receptor for Candida albicans that stimulates antifungal activity during oropharyngeal infection. Cell Rep 28:423–433. doi:10.1016/j.celrep.2019.06.020 31291578 PMC6638578

[64] Smeekens SP, Gresnigt MS, Becker KL, Cheng S-C, Netea SA, Jacobs L, Jansen T, van de Veerdonk FL, Williams DL, Joosten LAB, Dinarello CA, Netea MG. 2015. An anti-inflammatory property of Candida albicans β-glucan: Induction of high levels of interleukin-1 receptor antagonist via a dectin-1/CR3 independent mechanism. Cytokine 71:215–222. doi:10.1016/j.cyto.2014.10.013 25461401 PMC4437193

[65] Roeder A, Kirschning CJ, Schaller M, Weindl G, Wagner H, Korting HC, Rupec RA. 2004. Induction of nuclear factor- kappa B and c-Jun/activator protein-1 via toll-like receptor 2 in macrophages by antimycotic-treated Candida albicans. J Infect Dis 190:1318–1326. doi:10.1086/423854 15346344

[66] Pavlova A, Sharafutdinov I. 2020. Recognition of Candida albicans and role of innate type 17 immunity in oral candidiasis. Microorganisms 8:1340. doi:10.3390/microorganisms8091340 32887412 PMC7563233

[67] Wagner AS, Hancock TJ, Lumsdaine SW, Kauffman SJ, Mangrum MM, Phillips EK, Sparer TE, Reynolds TB. 2021. Activation of Cph1 causes ß(1,3)-glucan unmasking in Candida albicans and attenuates virulence in mice in a neutrophil-dependent manner. PLoS Pathog 17:e1009839. doi:10.1371/journal.ppat.1009839 34432857 PMC8423308

[68] Marakalala MJ, Vautier S, Potrykus J, Walker LA, Shepardson KM, Hopke A, Mora-Montes HM, Kerrigan A, Netea MG, Murray GI, Maccallum DM, Wheeler R, Munro CA, Gow NAR, Cramer RA, Brown AJP, Brown GD. 2013. Differential adaptation of Candida albicans in vivo modulates immune recognition by dectin-1. PLoS Pathog 9:e1003315. doi:10.1371/journal.ppat.1003315 23637604 PMC3630191

