# Supplementary material for: Loss of Fgr41 in Candida albicans attenuates virulence and increases proinflammatory immune responses in a manner that is dependent on β(1,3)-glucan but not dectin-1
Source: Infect Immun. 2026 Mar 27;94(4):e00523-25. doi: 10.1128/iai.00523-25 (PMC13081735; doi:10.1128/iai.00523-25)
Supplement: Supplemental material — Fig. S1 to S4; Tables S1 to S5. [file iai.00523-25-s0001.pdf]

SUPPLEMENTAL MATERIAL

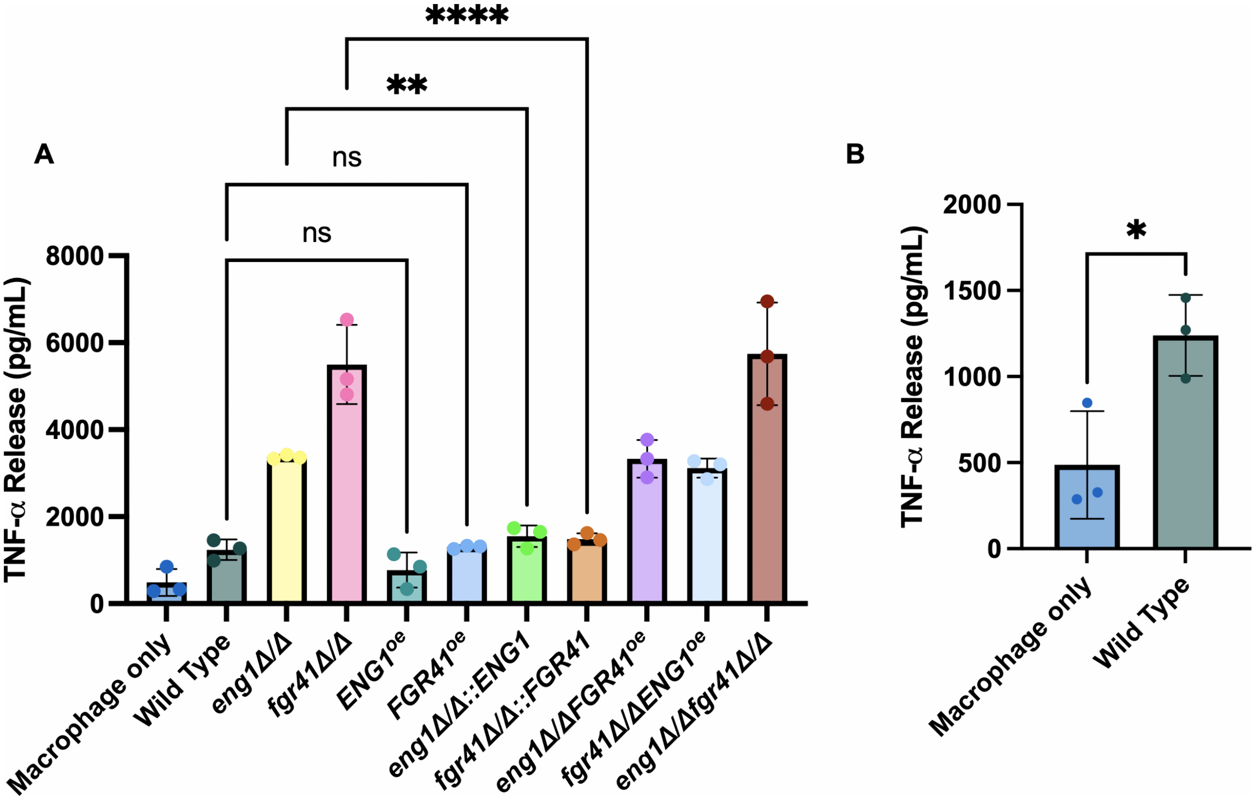

Figure S1. TNF-α release stimulated by *ENG1/FGR41* combination mutants. Data from Fig 1D including additional complement and overexpression strains. RAW264.7 macrophages and UV-inactivated *C. albicans* were co-incubated for 4 hours at 37°C + 5% CO_2. TNF-α concentrations in the supernatant were determined via ELISA. The experiment was performed three times with three technical replicates each time; each point represents the average of three technical replicates. (A) shows all strains tested, while (B) contains unchallenged macrophages and wild type analyzed in isolation, which revealed a significant difference. (****p < 0.0001, **p < 0.01, *p < 0.05, by one-way ANOVA (A) or Welch's *t*-test (B), ns = not significant).

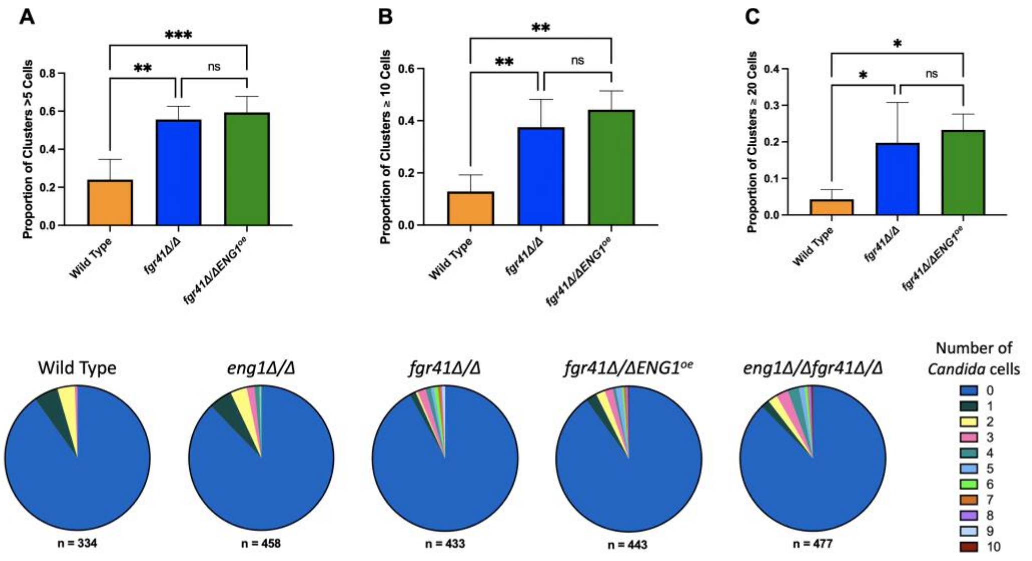

Figure S2. Average size of clusters in cell separation mutants and phagocytosis of *C. albicans* by macrophages. (A-C) Strains were grown to stationary phase before being washed and mounted for light microscopy. Cell clusters in a total of 72 fields of view were counted for each strain, over 4 biological replicates. (***$p < 0.001$, **$p < 0.01$, *$p < 0.05$, by one-way ANOVA, ns = not significant). (D) Quantification of the number of *C. albicans* cells contained within each macrophage. This data is also shown in Fig 2A, but here includes macrophages with no phagocytosed *C. albicans*. A total of 40 images were analyzed for each strain. *C. albicans* cells were counted as being phagocytosed if they appeared internal to the macrophage and/or caused a gap in green fluorescence. (n = the total number of macrophages counted).

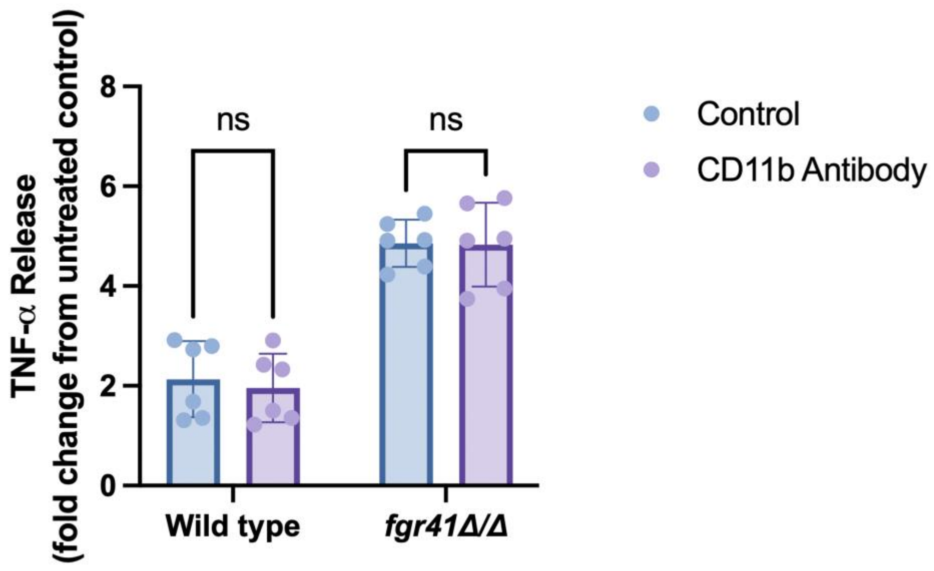

Figure S3. Effect of anti-CD11b antibody on TNF-α release stimulated by *eng1Δ/Δ* and *fgr41Δ/Δ* strains. RAW264.7 macrophages were treated with PBS or 10 ng/µl CD11b antibody for 1 hour prior to co-incubation with UV-inactivated *C. albicans*. Macrophages and *C. albicans* were co-incubated for 4 hours, and supernatant TNF-α concentrations were measured with an ELISA. The experiment was performed two times with three technical replicates each time. Significance was assessed using a one-way ANOVA (ns = not significant).

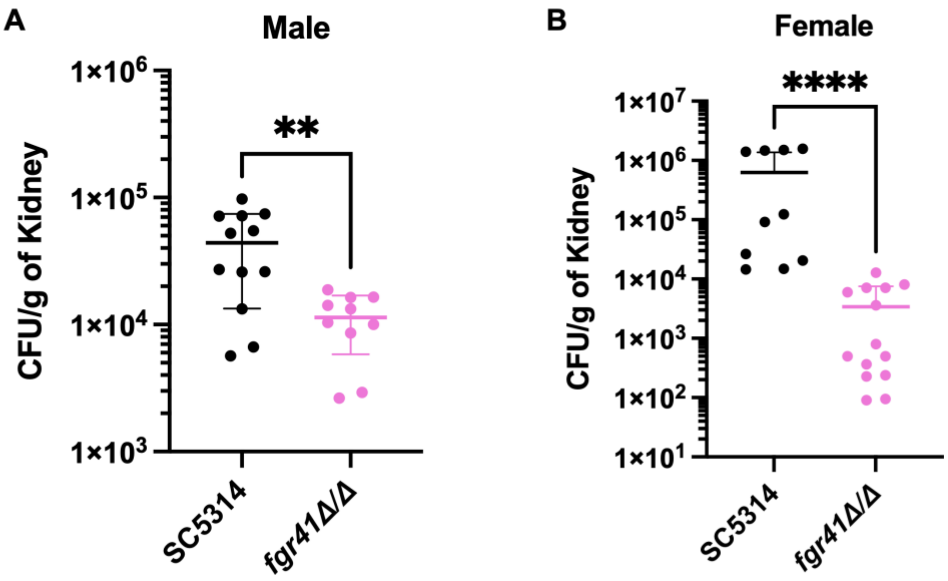

Figure S4. The *fgr41Δ/Δ* mutant causes reduced kidney fungal burden in C57BL/6J mice. Both male (A) and female (B) mice were injected with 2.5×10^6 cells of either wild type or *fgr41Δ/Δ C. albicans*. Kidneys were harvested at 2 dpi, homogenized, and plated for CFUs. Infection with the *fgr41Δ/Δ* strain led to reduced kidney fungal burden compared to wild type in both male and female mice. (****$p < 0.0001$, **$p < 0.01$, by Welch's *t*-test).

S1 Table. Quantification of 30 Minute Western Blots (Fig 4A-D).

	**IκBα**	**p100**	**p52**	**p38**	**phospho-p38**
**Wild Type**	1.396 ± 1.044	0.69 ± 0.147	0.935 ± 0.33	0.989 ± 0.09	1.575 ± 0.168
eng1Δ/Δ	1.06 ± 0.213	0.844 ± 0.144	1.002 ± 0.412	1.173 ± 0.573	2.89 ± 0.198*
fgr41Δ/Δ	1.715 ± 0.863	1.148 ± 0.088	1.324 ± 0.022	0.888 ± 0.219	3.374 ± 1.246**
**Stimulatory Laminarin**	1.143 ± 0.864	1.361 ± 0.251*	2.381 ± 0.362***	0.683 ± 0.237	7.04 ± 2.492***

Quantification of the relative fluorescence for all replicates of each Western blot in Fig 4A-D. The intensities of each band of interest were normalized to the loading control (tubulin), then converted to fold change from unstimulated macrophages (MΦ only). Shown is the average and standard deviation of the fold change across three biological replicates of each blot. Stars indicate a significant change over MΦ only (***p < 0.001, **p < 0.01, *p < 0.05, by one-way ANOVA).

S2 Table. Quantification of 4 Hour Western Blots (Fig 4E-H).

	**IκBα**	**p100**	**p52**	**p38**	**phospho-p38**
**Wild Type**	1.004	0.816	0.697	0.750	1.127
eng1Δ/Δ	0.985	0.638	0.707	0.884	0.781
fgr41Δ/Δ	1.027	0.668	0.822	0.917	0.792
**Stimulatory Laminarin**	0.853	0.736	1.387	1.065	2.622

Quantification of the relative fluorescence for each Western blot in Fig 4E-H. The intensities of each band of interest were normalized to the loading control (tubulin), then converted to fold change from unstimulated macrophages.

S3 Table. Strains used in this study.

Strain	Name	Genotype	Parent	Reference
Wild Type	AWY006	*LEU2/leu2Δ*	SC5314	(1)
eng1Δ/Δ	AKY046	*eng1Δ/Δ LEU2/leu2Δ*	AWY006	This study
fgr41Δ/Δ	SLY005	*fgr41Δ/Δ LEU2/leu2Δ*	AWY006	(1)
ENG1^{oe}	AKY007	P_{ENO1}*-ENG1-SAT1 LEU2/leu2Δ*	AWY006	This study
FGR41^{oe}	SLY015	P_{ENO1}*-FGR41-SAT1 LEU2/leu2Δ*	AWY006	(1)
eng1Δ/Δ::ENG1	AKY053	*eng1Δ/Δ* P_{ENO1}*-ENG1-SAT1 LEU2/leu2Δ*	AKY046	This study
fgr41Δ/Δ::FGR41	SLY013	*fgr41Δ/Δ* P_{ENO1}*-FGR41-SAT1-LEU2/leu2Δ*	SLY005	(1)
eng1Δ/ΔFGR41^{oe}	AKY047	*eng1Δ/Δ* P_{ENO1}*-FGR41-SAT1 LEU2/leu2Δ*	AKY046	This study
fgr41Δ/ΔENG1^{oe}	AKY013	*fgr41Δ/Δ* P_{ENO1}*-ENG1-SAT1-LEU2/leu2Δ*	SLY005	This study
eng1Δ/Δfgr41Δ/Δ	AKY049	*eng1Δ/Δ fgr41Δ/Δ LEU2/leu2Δ*	SLY005	This study

S4 Table. Plasmids used in this study.

Name	Description	Markers	Reference
pBT1	P_{ENO1}	Ampicillin, Nourseothricin	(2)
pAEK012	P_{ENO1}-*ENG1*	Ampicillin, Nourseothricin	This study
pSL003	P_{ENO1}-*FGR41*	Ampicillin, Nourseothricin	(1)
CaHygB-flipper	CaHygB Flipper	Ampicillin, Hygromycin	(3)
pBSS2	SAT1 Flipper	Ampicillin, Nourseothricin	(3)

S5 Table. Primers used in this study.

Name	Sequence	Description
AKO1	AAAAGCGGCCGCCGCTATGCTTTTC AAATCCG	Forward primer for *ENG1* amplification with Not1 cut site
AKO2	AAAAGCGGCCGCTTGATTGCAGTG GCATTTTT	Reverse primer for *ENG1* amplification with Not1 cut site
BTO33	GACTAACGATTTCTATAAGG	Forward check primer in P_{ENO1} for cloning into pBT1 and colony PCR for *C. albicans* integration
AKO3	GTTGTCTTTAAAGTCACAGCACC	*ENG1* reverse check primer for cloning into pBT1 and colony PCR for *C. albicans* integration
AWO363	GAGAGTGACAGTTGTAGTGATTG	*FGR41* reverse check primer for colony PCR
AKO57	CAATGAAATACAAAAAAAAAAGCA AAAAACTTTTCCGGAATTAGAAGG GTTTTCCCAGTCACGACGT	*ENG1* CRISPR-Cas9 repair template forward (ENG1CC9KOF) with homology to pBSS2/pHygR
AKO58	GCACCAGCAGCAAAAGCCAAACTC CAAGTTCTACTTTGACCATTATCTA AGTGTGGAATTGTGAGCGGAT	*ENG1* CRISPR-Cas9 repair template reverse (ENG1CC9KOR) with homology to pBSS2/pHygR

AWO413	GGTTGTTAAACGATAAACAATC	ENG1DETf- forward check primer for CRISPR deletion
AWO414	GAGTCATTGGAAGTTGAGTCC	ENG1DETR- reverse check primer for CRISPR deletion
crENG1up	GCATCAGATTGAGCGTGTTC	Upstream crRNA for ENG1 deletion
crENG1down	CAAATAACTGGGATGACAAG	Downstream crRNA for ENG1 deletion

REFERENCES

1. Wagner AS, Lumsdaine SW, Mangrum MM, King AE, Hancock TJ, Sparer TE, Reynolds TB. 2022. Cek1 regulates β(1,3)-glucan exposure through calcineurin effectors in Candida albicans. PLoS Genet 18:e1010405.
2. Tams RN, Cassilly CD, Anaokar S, Brewer WT, Dinsmore JT, Chen YL, Patton-Vogt J, Reynolds TB. 2019. Overproduction of Phospholipids by the Kennedy Pathway Leads to Hypervirulence in Candida albicans. Front Microbiol 10:86.
3. Liu J, Vogel AK, Miao J, Carnahan JA, Lowes DJ, Rybak JM, Peters BM. 2022. Rapid Hypothesis Testing in Candida albicans Clinical Isolates Using a Cloning-Free, Modular, and Recyclable System for CRISPR-Cas9 Mediated Mutant and Revertant Construction. Microbiol Spectr 10:e0263021.
